# Collagen XVII inhibits breast cancer cell proliferation and growth through deactivation of the AKT/mTOR signaling pathway

**DOI:** 10.1371/journal.pone.0255179

**Published:** 2021-07-22

**Authors:** Muttarin Lothong, Watchara Sakares, Pornchai Rojsitthisak, Chizu Tanikawa, Koichi Matsuda, Varalee Yodsurang

**Affiliations:** 1 Faculty of Pharmaceutical Sciences, Department of Pharmacology and Physiology, Chulalongkorn University, Bangkok, Thailand; 2 Natural Products for Ageing and Chronic Diseases Research Unit, Chulalongkorn University, Bangkok, Thailand; 3 Faculty of Pharmaceutical Sciences, Department of Food and Pharmaceutical Chemistry, Chulalongkorn University, Bangkok, Thailand; 4 Laboratory of Genome Technology, Human Genome Center, The Institute of Medical Science, The University of Tokyo, Tokyo, Japan; 5 Department of Computational Biology and Medical Sciences, Laboratory of Clinical Genome Sequencing, Graduate School of Frontier Sciences, The University of Tokyo, Tokyo, Japan; 6 Preclinical Toxicity and Efficacy Assessment of Medicines and Chemicals Research Cluster, Chulalongkorn University, Bangkok, Thailand; University of Bergen, NORWAY

## Abstract

Collagen XVII (COL17), a cell-matrix adhesion protein, has been found to be suppressed in breast cancer. Our previous data demonstrated a preventive role of COL17 in breast cancer invasiveness. The present study used the stable COL17-overexpressing MCF7 and MDA-MB-231 cells to reveal an anti-proliferative effect of COL17 on breast cancer cell through mTOR deactivation. Cell proliferation was negatively correlated with the expression level of COL17 in a concentration-dependent manner in both conventional and three-dimensional (3D) culture systems. The correlation was confirmed by decreased expression of the proliferative marker Ki67 in COL17-expressing cells. In addition, overexpression of COL17 reduced the clonogenicity and growth of the cells. We demonstrated that COL17 affects the AKT/mTOR signaling pathway by deactivation of AKT, mTOR and downstream effectors, particularly 4EBP1. Moreover, mice xenografted with high COL17-expressing cells exhibited delayed tumor progression and prolonged survival time. The high expression of *COL17A1* gene encoding COL17 is associated with low-proliferation tumors, extended tumor-free period, and overall survival of breast cancer patients. In conclusion, our results revealed the novel function of COL17 using *in vitro* and *in vivo* models and elucidated the related pathway in breast cancer cell growth and proliferation.

## Introduction

Breast cancer is the most common cancer in women and one of the leading causes of cancer death [1]. It is characterized by the uncontrolled growth of abnormal cells in the glands or ducts of mammary tissues [2]. Extensive research related to the molecular features and oncogenic pathways of breast cancer is continuously underway to identify new targets for breast cancer treatment. It is of interest that the tumor microenvironment is involved in tumor progression and invasiveness [3,4]. Accordingly, the biochemical mechanisms of extracellular matrix molecules associated with cancer cell development and metastasis have been addressed [3].

Hemidesmosomes (HDs) are specialized structures that are composed of multiple proteins facilitating the stable attachment of basal epithelial cells to the underlying basement membrane. Two types of HDs are classified on the basis of their distinct protein components. Type I HDs, consisting of integrin α6β4, plectin, tetraspanin, BP230 and collagen type XVII (COL17), are observed in stratified and pseudostratified epithelia such as skin and myoepithelial cells in the mammary gland [5]. However, type II HDs lacking BP230 and COL17 are found in simple epithelia such as the intestine [6]. Aberrant expression of hemidesmosomal proteins has been reported to be associated with the development and progression of several cancers [7,8].

COL17, a transmembrane protein encoded by the *COL17A1* gene, is a structural component of type I HDs and plays a critical role in cellular adhesion to the underlying extracellular matrix [7]. A 120-kD extracellular domain of COL17, shed from a 180-KD full-length protein by a metalloproteinase, has various functions in normal tissues, including cell motility, cell differentiation, and skin inflammation [9,10]. Therefore, *COL17A1* mutations and autoantibodies against COL17 can cause blistering skin diseases caused by a loss of attachment between the epidermis and the underlying basement membrane [11,12]. Abnormal expression of *COL17A1* has been observed in several types of cancer, including breast, cervical, colorectal, and lung cancers [13–15]. In breast cancer, *COL17A1* is suppressed by DNA methylation and inactivation of p53 [15,16]. With regard to our previous research showing that COL17 can suppress breast cancer migration and invasion [16], in the present study, we further investigated the additional role of COL17 in breast cancer cell growth and proliferation and elucidated its regulatory pathway using COL17-overexpressing MCF7 and MDA-MB-231 cells.

## Results

### Induction of COL17 expression by doxycycline

**Fig 1:**
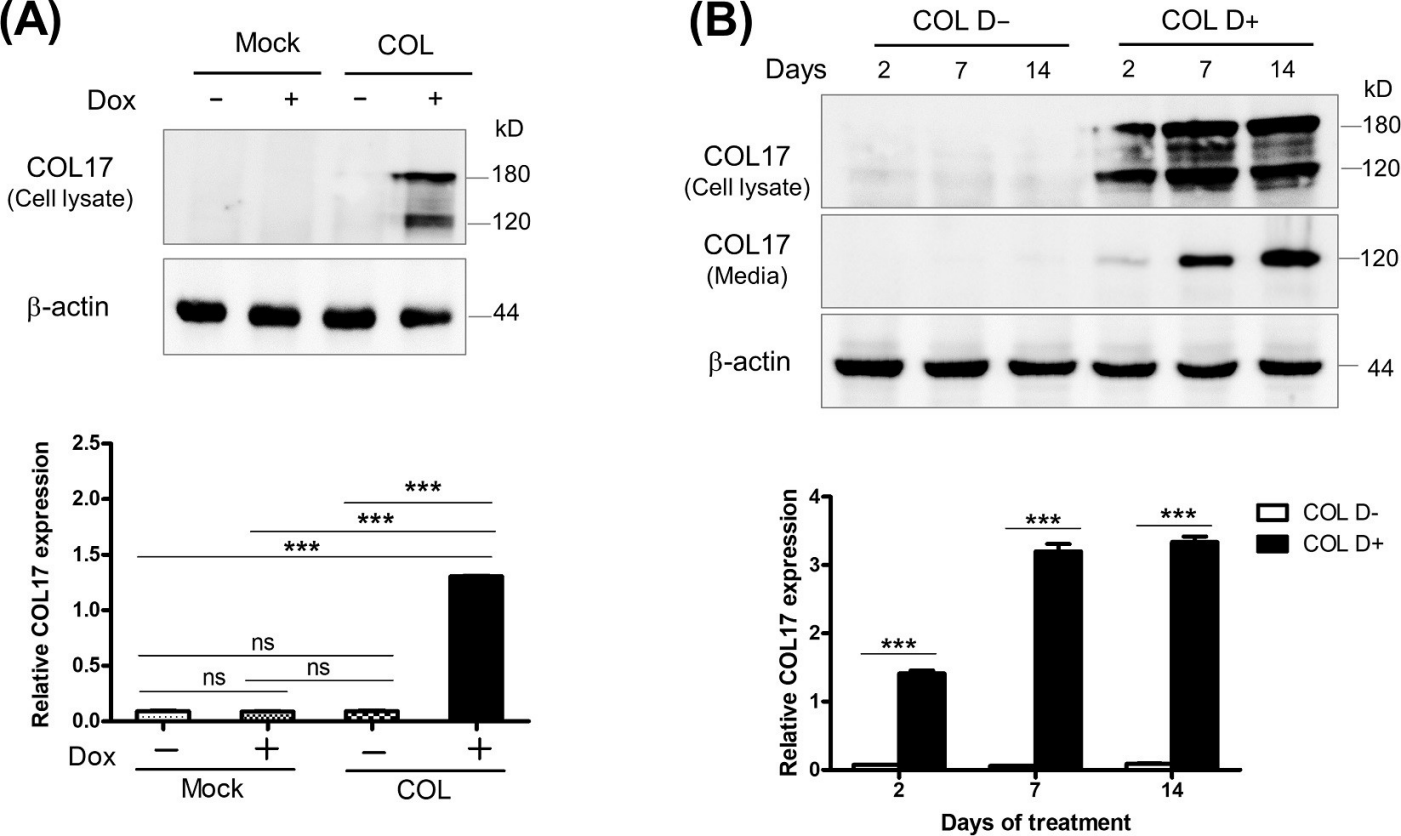
Induction of COL17 expression by doxycycline in MCF7/COL cells. The stable COL17-overexpressing MCF7/COL (COL) and mock cells were treated with 0.1 μg/ml of doxycycline (Dox, D+) or not treated (D−). **(A–B)** Western blot images of COL17 in COL and mock cells treated as indicated. The anti-collagen XVII (ab184996) and HRP-conjugated secondary antibodies (ab205718) were used for blotting. **(A)** Relative COL17 expression in COL and mock cells treated with Dox for 2 days. **(B)** Relative COL17 expression in COL cells treated with Dox for 2, 7 and 14 days. Proteins in cell lysate and media were extracted from the same culture plate. The relative expression of the combined 180-kD and 120-kD forms of COL17 in cell lysate and media is quantified by ImageJ and normalized to β-actin (*n* = 3 technical replicates). Two-tailed Student’s *t*-test; ***P < 0.0001; ns, *P* ≥ 0.05.

To investigate the COL17 function in cell proliferation of primary breast cancer, a low invasive MCF-7 cell was genetically engineered to be MCF7/COL (COL) cells stably expressing COL17 in the presence of doxycycline (Dox) and control mock cells using tetracycline-regulated lentiviral expression system (S1 Fig). In the absence of Dox, COL17 was hardly detectable in mock and COL cells (Fig 1A). After Dox treatment, COL cells expressed the 180-kD full-length form and the 120-kD extracellular C-terminal domain (ectodomain) of COL17, whereas COL17 expression was not induced in mock cells. Induction of COL17 expression by Dox increased with time (Fig 1B). In Dox-treated COL, the COL17 level increased approximately 2.3- and 2.4-fold after 7- and 14-day treatment periods, respectively, compared with after a 2-day treatment period. The maximum induced expression of the combined two forms of COL17 detected at day 14 was 38-fold greater than that in untreated COL cells.

### COL17 inhibits the cell proliferation, clonogenicity and growth in 2D culture

**Fig 2:**
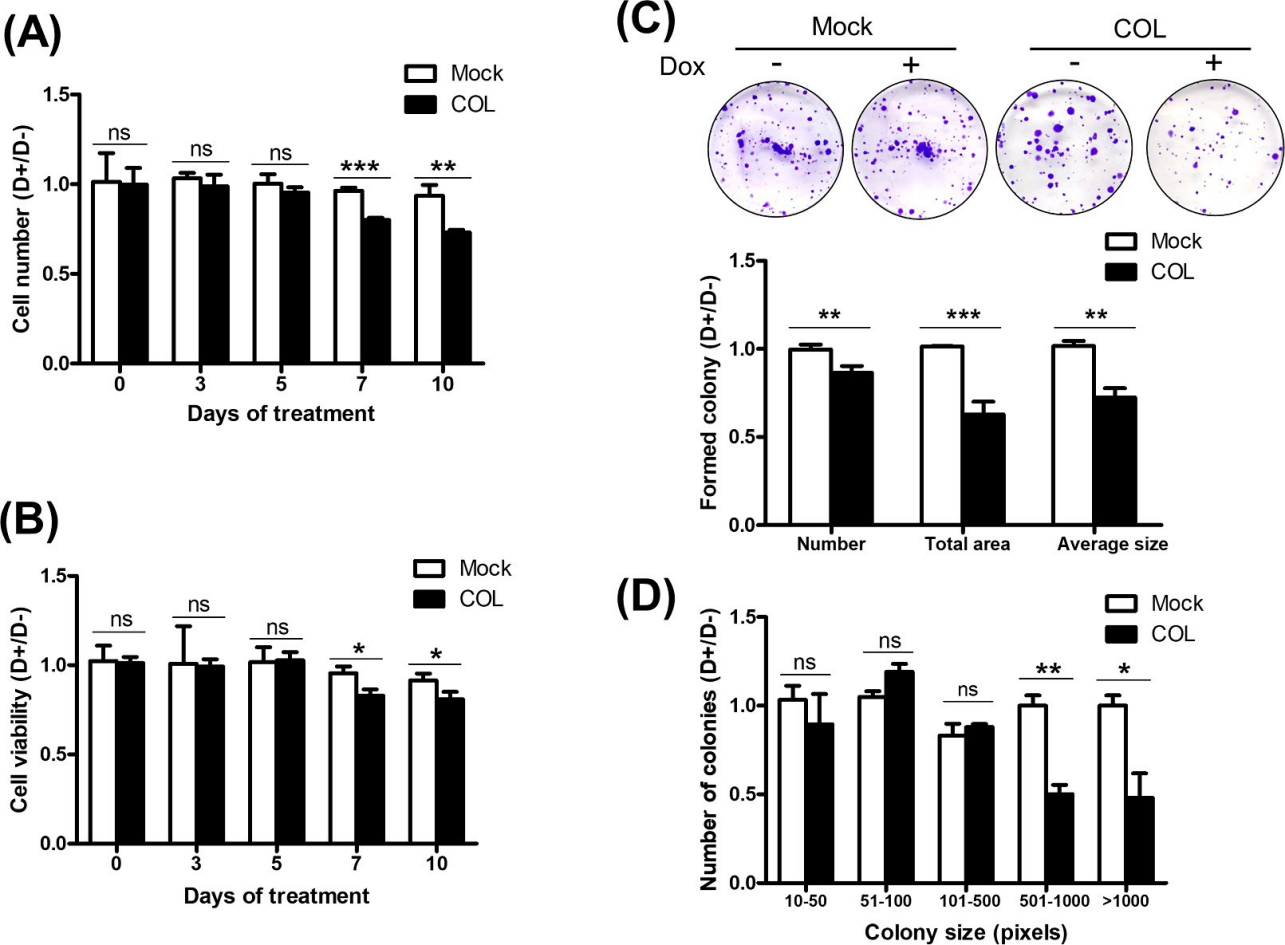
COL17 inhibits the cell proliferation, clonogenicity and growth in 2D culture. **(A–B)** MCF7/COL (COL) and mock cells were grown in 96-well plate for 48 hours and then treated with 0.1 μg/ml of doxycycline (Dox, D+) or not treated (D−). Cell numbers and cell viability were observed before and at 3, 5, 7 and 10 days of Dox treatment (*n* = 5 biological replicates). The results of D+ were normalized to those of D− groups. **(A)** The number of COL and mock cells were determined by cell counting. **(B)** Cell viability of COL and mock was determined by MTT assay. **(C–D)** The COL and mock cells were grown in 6-well plate and treated as **A–B**. The represented images of colony formation assay of COL and mock cells observed at 12 days of Dox treatment. The images of cell colonies were analyzed by ImageJ program. Number of colonies, total area, and average size of colonies of D+ were normalized with those of D− groups (*n* = 3 biological replicates). Two-tailed Student’s *t*-test; *P < 0.05, **P < 0.01, ***P < 0.0001; ns, *P* ≥ 0.05.

The proliferation of the stable MCF7/COL and mock cells was examined after up to 10 days of Dox treatment using cell counting and MTT assays. Because there was baseline expression of COL17 in untreated COL cells (Fig 1B), resulted from incomplete suppression by Tet repressor (S1 Fig), the net effect of COL17 is displayed as the assay results of treated cells normalized to those of untreated cells. The relative results of the COL group were then compared to those of the mock group. At 3 or 5 days of treatment, the numbers of COL and mock cells were not affected by Dox (Figs 2A and S2). However, the number of Dox-treated COL cells was markedly decreased 0.8- and 0.7-fold on days 7 and 10, respectively (Fig 2A). Whereas the relative number of mock cells was not affected by Dox throughout the experiment. Consistent with the change in the cell number, the cell viability of the Dox-treated COL cells significantly decreased after 7 and 10 days of treatment (Fig 2B). The overall results indicated that COL17 expression induced by 7- and 10-day Dox treatment significantly suppressed the number and cell viability of MCF7 cells in 2D culture.

To verify that the reductions in the number and viability of COL17-expressing cells did not triggered by apoptosis or necrosis at the late culture period, the cells were stained with Hoechst 33342 and a percentage of cell death was calculated. S3 Fig showed that there were less than 10% of cell death in both mock and COL cells at 10 days of Dox treatment. There was no statistical difference in percentage of cell death for all conditions. This result demonstrates that the inhibitory effect of COL17 on the cell number is mainly related to proliferation rather than cell death.

To study clonogenicity, a colony formation assay was performed after 12 days of Dox treatment. There were significant reductions in the number of colonies (0.86-fold), total area (0.63-fold), and average size of colonies (0.72-fold) of COL17-expressing cells (Fig 2C). To gain insight into the effect of COL17 on the size of the affected colonies, the numbers of colonies of various sizes were analyzed (Fig 2D). The number of small colonies (10–50, 51–100, and 101–500 pixels) was not different between COL17-expressing cells and nonexpressing cells. Interestingly, the number of COL17-expressing cells with large colonies (both 501–1000 and >1000 pixels) was reduced nearly 50% compared to that of mock cells with large colonies, demonstrating the negative relationship between COL17 expression and cell growth. These results indicated that overexpression of COL17 decreased the proliferation, clonogenicity and growth of MCF7 cells.

### COL17 effect on Ki67 reduction

**Fig 3:**
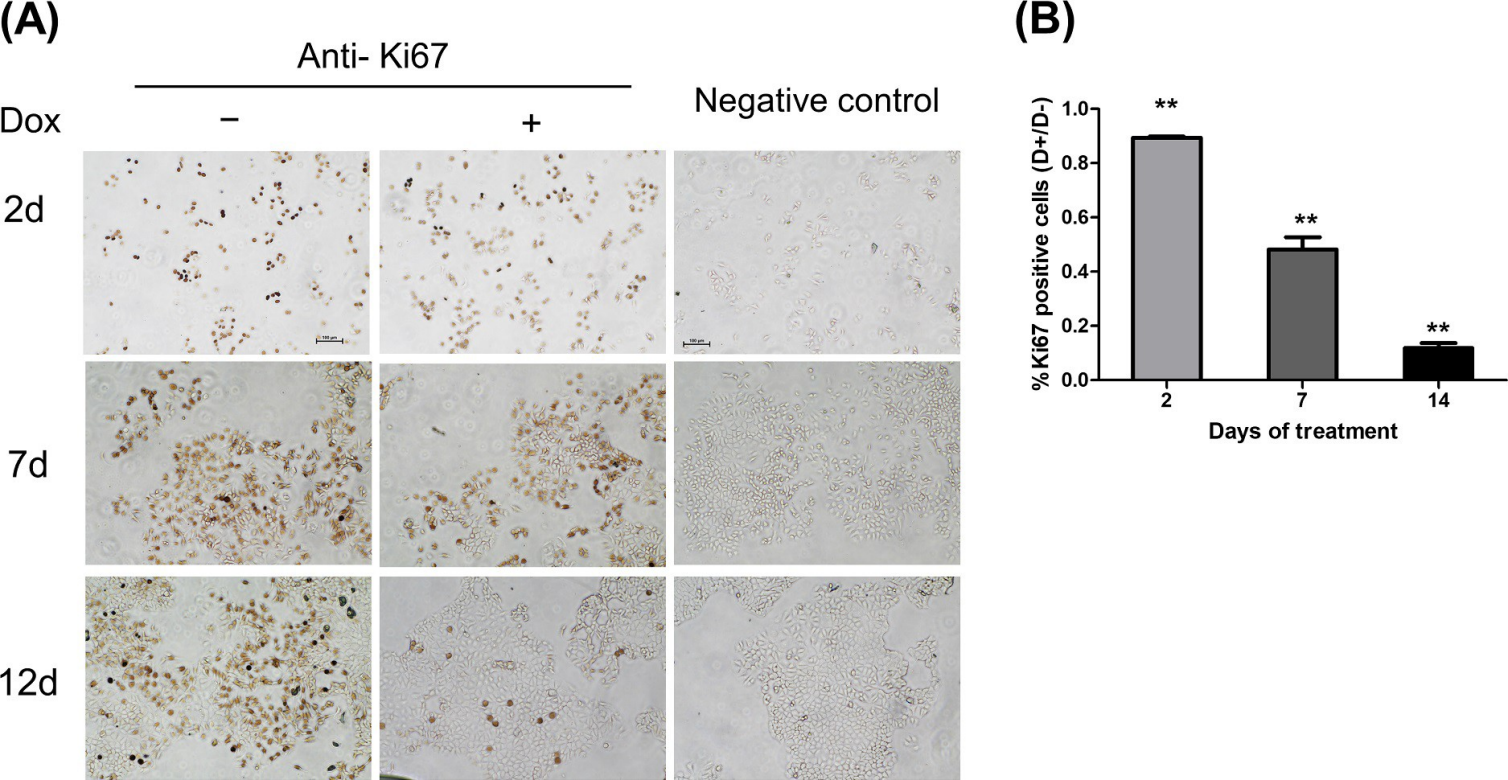
COL17 effect on Ki67 reduction. MCF7/COL cells were grown in 24 well-plate and treated with 0.1 μg/ml of doxycycline (Dox, D+) or not treated (D−). Cells were fixed and subjected to immunocytochemistry to observed Ki67 expression at 2, 7, and 14 days of treatment. (*n* = 3 biological replicates). **(A)** The time-course representative images of COL cells blotted with anti-Ki67 or without primary antibody as negative controls. **(B)** The number of Ki67-positive COL cells was quantified by ImageJ software. The percentage of Ki-67 positive area was normalized by the total number of cells (3 independent areas per sample, 3 samples per group), and displayed as ratio of the normalized expression between D+ and D− groups. Two-tailed Student’s *t*-test; **P < 0.01, ***P < 0.0001; ns, *P* ≥ 0.05.

The cellular expression of the proliferative marker Ki67 in stable MCF7/COL cells was evaluated after up to 12 days of Dox treatment. Following the induction of COL17, the percentage of cells expressing Ki67 decreased to 0.89-fold of the non-COL17-expressing cells on day 2 (Fig 3A and 3B) and gradually declined to 0.48- and 0.12-fold on days 7 and 12, respectively. The data demonstrated the association between COL17 overexpression and reduced Ki67 expression, confirming that COL17 inhibited the proliferation of MCF7 cells in a concentration-dependent manner.

### COL17 reduces the spheroid size and proliferation in 3D culture

**Fig 4:**
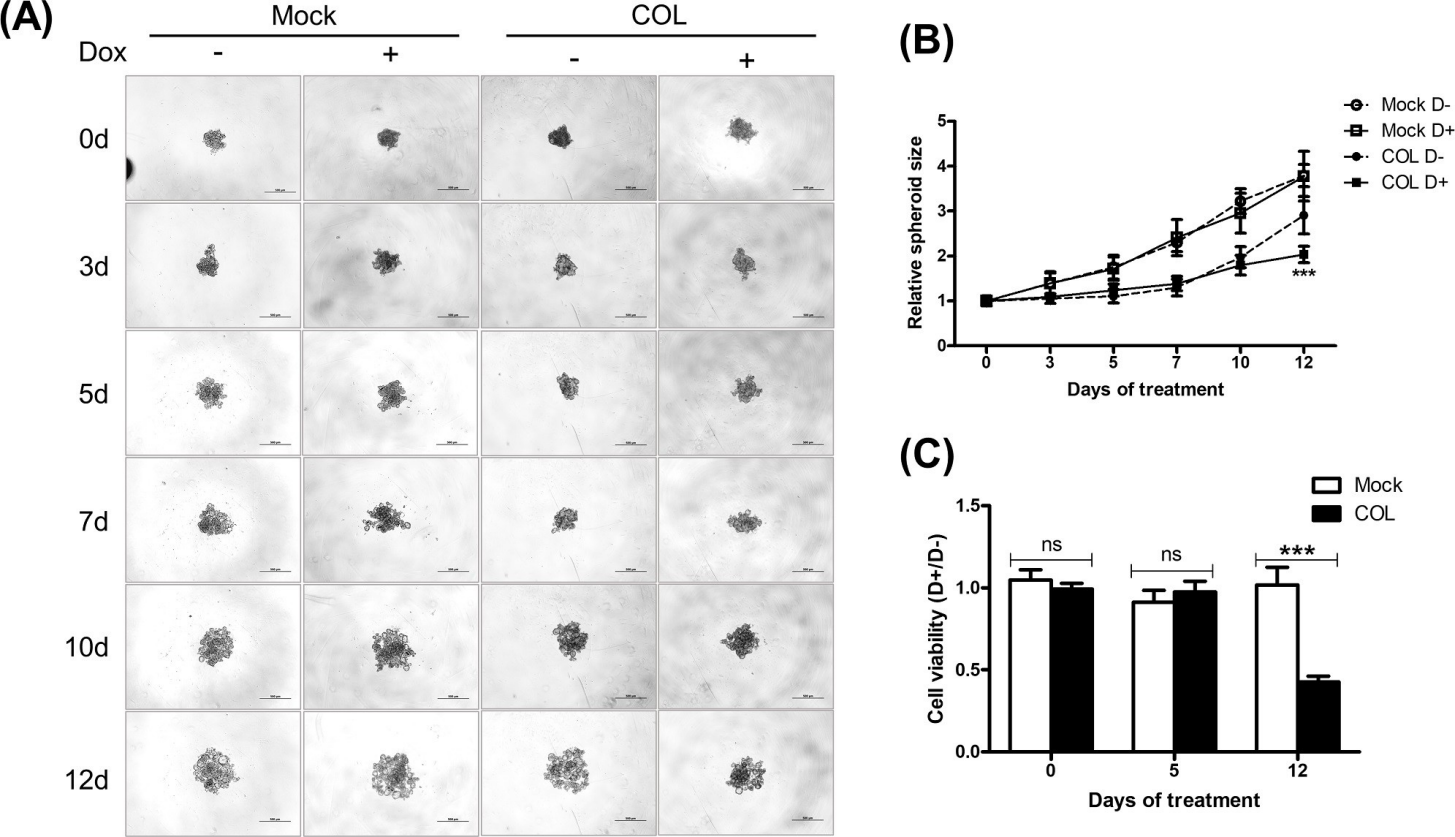
COL17 reduces the spheroid size and proliferation in 3D culture. MCF7/COL (COL) and mock cells were grown in ultra-low attachment 96-well plate for 48 hours and then treated with 0.1 μg/ml of doxycycline (Dox, D+) or not treated (D−). **(A)** The time-course representative images of MCF7 spheroids before and at 3, 5, 7, 10 and 12 days of Dox treatment. The images were taken by a phase-contrast microscope at 10× magnification. The scale bars indicate 500-μm length. **(B)** The spheroid area, excluding the intra-spheroid spaces, was measured by ImageJ software and displayed as spheroid size at 3, 5, 7, 10 and 12 days of treatment, relative to that before the treatment (*n* = 5 biological replicates). The statistical difference was compared between treated and untreated cells at each timepoint. Two-tailed Student’s *t*-test, ***P < 0.0001; no star, *P* ≥ 0.05. **(C)** Cell viability of COL and mock spheroids was evaluated by 3D ATP assay at 0, 5, and 12 days of treatment (*n* = 5 biological replicates). The ratio of cell viability between D+ and D− groups is shown. Two-tailed Student’s *t*-test, ***P < 0.0001; ns, *P* ≥ 0.05.

To mimic tumor formation *in vivo*, stable MCF7/COL and mock cells were cultured in a 3D culture system. After allowing aggregation for 48 hours, the spheroids were treated with Dox. Single spheroids in individual wells were examined before and after 3, 5, 7, 10, and 12 days of Dox treatment (Fig 4A). The relative spheroid size of mock cells did not change after Dox treatment for any time period, indicating that Dox alone has no effect on spheroid formation (Fig 4B). On day 12, the non-COL17-producing cells formed large loosely packed spheroids with intercellular spaces, but the COL17-expressing cells formed tightly packed spheroids (Fig 4A) that were significantly smaller than those formed by non-COL17-producing cells (Fig 4B). The results suggested that the long-term induction of COL17 affected spheroid formation and decreased the size of MCF7 spheroids.

The effects of COL17 on the number of viable cells in spheroids determined using the ATP assay are shown in Fig 4C. On day 12, the Dox-treated COL cells exhibited more than 50% lower cell viability than the untreated cells. As expected, Dox did not alter the cell number of mock spheroids at any time point. The results revealed that the 12-day induction of COL17 expression reduced the spheroid size and the proliferation of MCF7 cells in 3D culture.

### COL17 deactivates the AKT/mTOR signaling pathway

**Fig 5:**
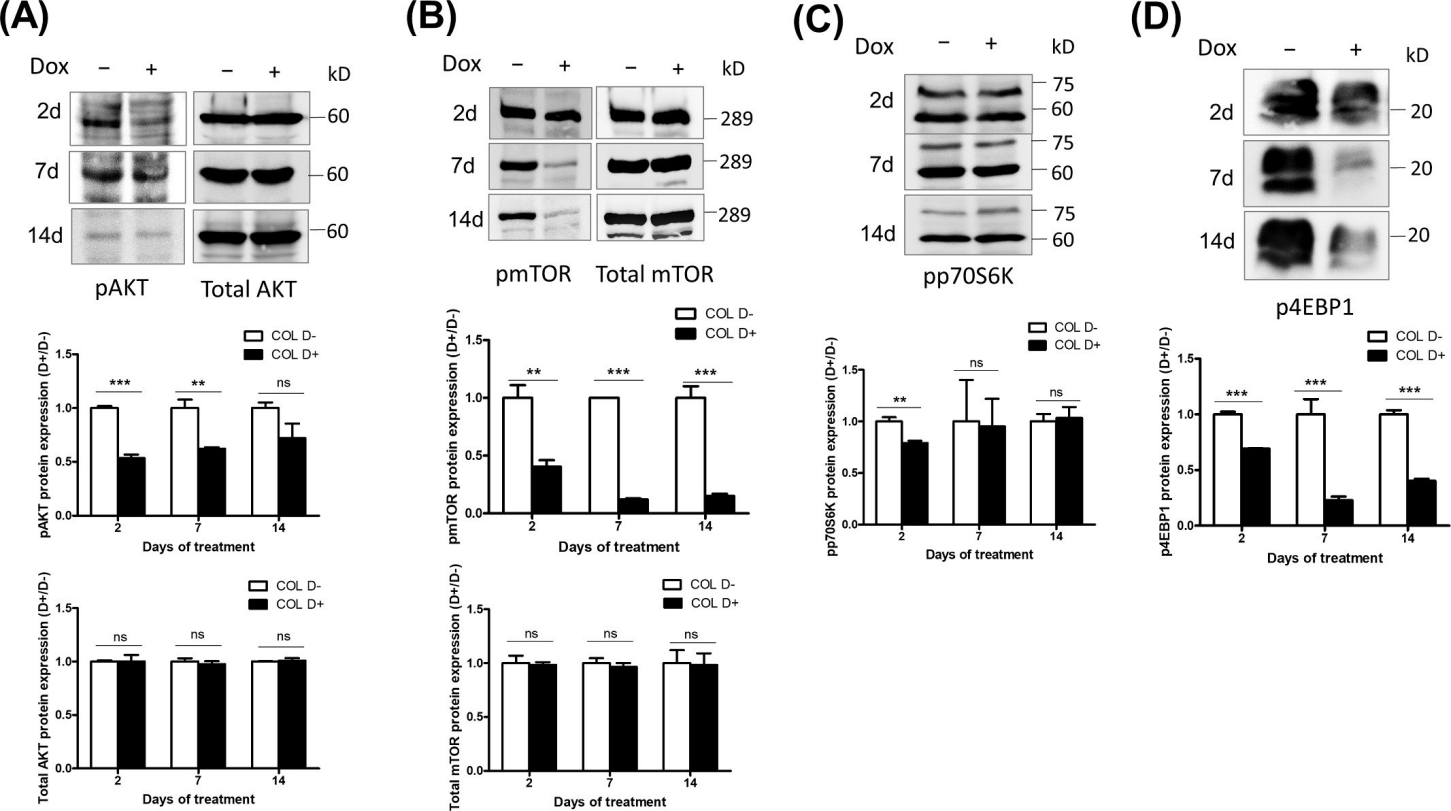
COL17 deactivates an AKT/mTOR signaling pathway. **(A–D)** MCF7/COL cells were not treated (D−) or treated with 0.1 μg/ml of doxycycline (Dox, D+). Total protein was collected at 2, 7 and 14 days of Dox treatment. Protein expressions of AKT/mTOR signaling molecules were analyzed using western blotting. The band intensity was measured by ImageJ software, normalized to β-actin (represented in Fig 1B), and displayed as a ratio of the normalized expression between D+ and D− groups (*n* = 3 technical replicates). The statistical difference between D+ and D− groups was analyzed using two-tailed Student’s *t*-test; *P < 0.05 **P < 0.01, ***P < 0.0001; ns, *P* ≥ 0.05. pAKT, phosphorylated AKT (Ser 473); pmTOR, phosphorylated mTOR (Ser 2448); pp70S6K, phosphorylated p-70 S6 kinase (Thr389); p4EBP1, phosphorylated 4EBP1 (Thr37/46).

Considering the common alterations in AKT signaling molecules found in breast cancers and as their use as targets of anticancer drugs [17,18], the AKT/mTOR pathway was further investigated to determine whether it was involved in the inhibition of breast cancer progression by COL17. The protein levels of AKT, mTOR, p70S6K, 4EBP1, and their phosphorylated forms were evaluated in MCF7/COL cells after 2, 7 and 14 days of Dox treatment. The overexpression of COL17 did not alter the levels of AKT and mTOR at any time point (Fig 5A and 5B). However, decreased phosphorylation of AKT, mTOR, p70S6K, and 4EBP1 was detected in COL17-expressing cells at different time points, indicating deactivation of the AKT/mTOR pathway (Fig 5A–5D). The phosphorylation of mTOR and 4EBP1 was markedly decreased at all time points, particularly on day 7, at which point the levels were reduced 0.12- and 0.23-fold, respectively (Fig 5B and 5D). The level of pAKT was significantly decreased approximately 0.5- and 0.6-fold after 2 and 7 days of treatment, respectively (Fig 5A). phosphorylation of p70S6K slightly decreased 0.89-fold after 2 days of treatment (Fig 5C). These results revealed that COL17 deactivated the AKT/mTOR signaling pathway in MCF7 cells by inhibiting the phosphorylation of molecules responsible for cell growth and proliferation.

### COL17 delays breast cancer progression and prolongs survival in mice

To evaluate the effects of COL17 on the overall survival of mice in addition to the progression of primary tumor, an MDA-MB-231 cells were used for a xenograft. The genetically engineered COL17-overexpressing MDA-MB-231 (MDA/COL) cells, which have been shown to exhibit reduced cell invasiveness *in vitro* [16], were grafted into mice. MDA/COL and mock cells were pretreated with Dox for 48 hours and implanted into two sites in the inguinal mammary fat pads of each mouse (4 mice per group). COL17 expression in xenografted tissues induced by treating the mice with Dox in drinking water was confirmed by western blot analysis of the primary tumors at the end of the study (S4 Fig). Similar to MCF7/COL cells, MDA/COL cells stably expressed COL17 in the presence of Dox.

**Fig 6:**
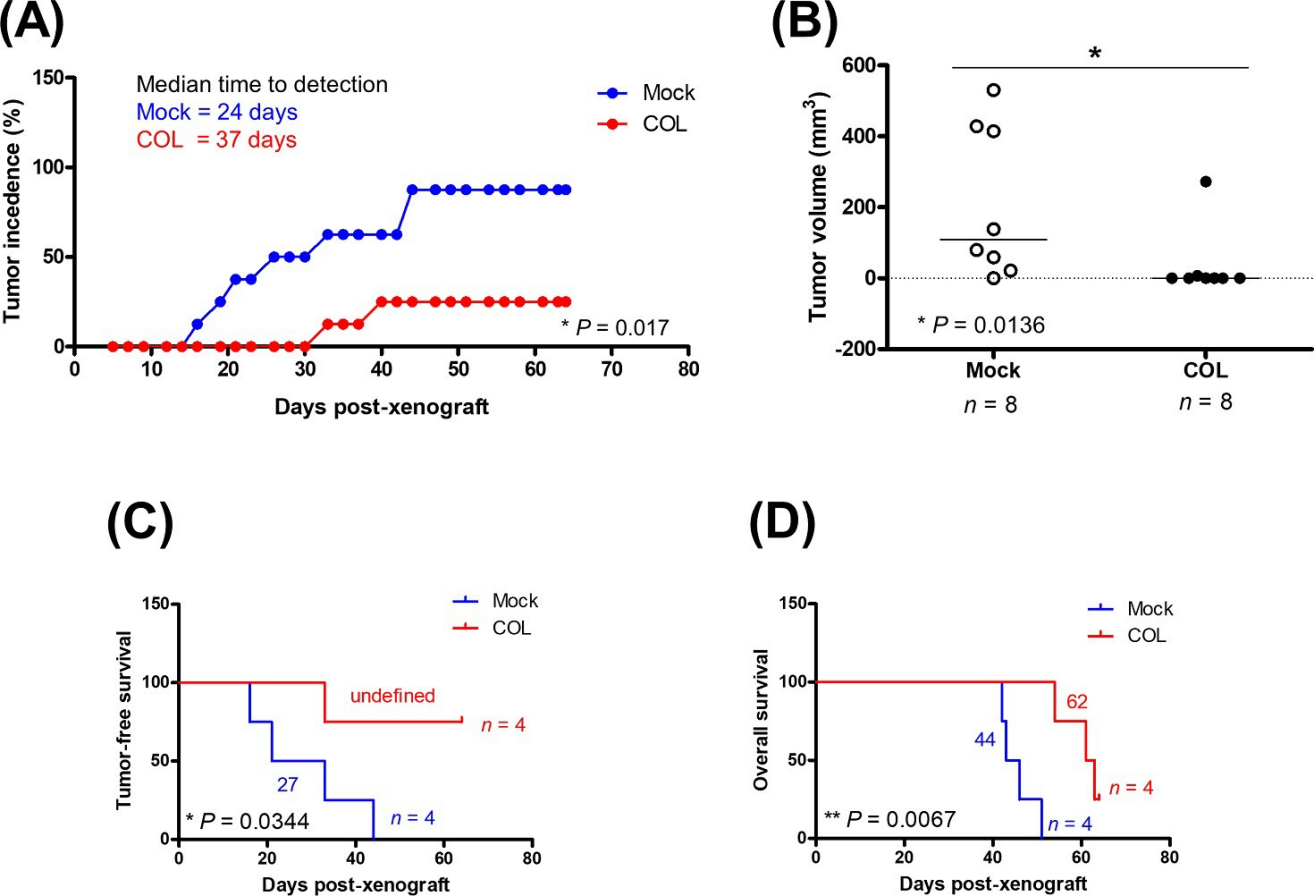
COL17 delays breast cancer progression and prolongs survival in mice. The stable MDA-MB-231/COL17 (COL) and mock cells were treated with 0.1 μg/ml of doxycycline (Dox) for 48 hours before implanting into mammary fat pads of the mice (2 sites per mouse, 4 mice per group). The COL17 expression was induced by treating the mice with 1mg/ml Dox in drinking water. **(A)** The number of palpable tumors was displayed as a percentage of 8 implantation sites in mock (blue) and COL group (red). Median time-to-tumor detection in each group is presented. The difference of tumor incidences between COL and mock groups was analyzed by the two-tailed Student’s *t*-test. **(B)** The maximum volume of tumors measured at the end of study in mice grafted with mock or COL cell. Tumor-free survival **(C)** and overall survival **(D)** of xenograft mice were observed until the end of study (9-week post-xenograft). Log-rank test was used for survival analysis. Student’s *t*-test with Welch’s correction was used for boxplot. *P < 0.05, **P < 0.01.

Primary tumors were observed in all four mock-grafted mice, with an 87.5% tumor incidence (7 out of 8 sites in total), and most of the mice had detectable tumors at both injection sites (Fig 6A). In contrast, the primary tumors were detected in 2 out of 8 sites grafted with COL17-expressing cells (25% tumor incidence, *P* = 0.017 compared to mock) throughout nine weeks of experimentation. The time-to-tumor detection of the COL group was delayed to 37 days, whereas that of the mock group was 24 days post-xenograft (Fig 6A). The maximum size of tumor detected in this experiment was 530 mm^3^. The average size of the tumors in the COL group was significantly reduced compared to that in the mock group (35 vs 209 mm^3^, respectively, Fig 6B). All mice in the mock group met the humane endpoint and were euthanized prior to nine weeks of experiment. Whereas one mouse in the COL group was euthanized at the end of experiment. Moreover, the tumor-free and overall survival of the COL-grafted mice were significantly prolonged compared to those of the mock group (Fig 6C and 6D). The results suggested that the overexpression of COL17 limited the size of primary tumors, leading to delayed progression and better prognosis.

### High *COL17A1* level is associated with low proliferation and extended tumor progression in patients

**Fig 7:**
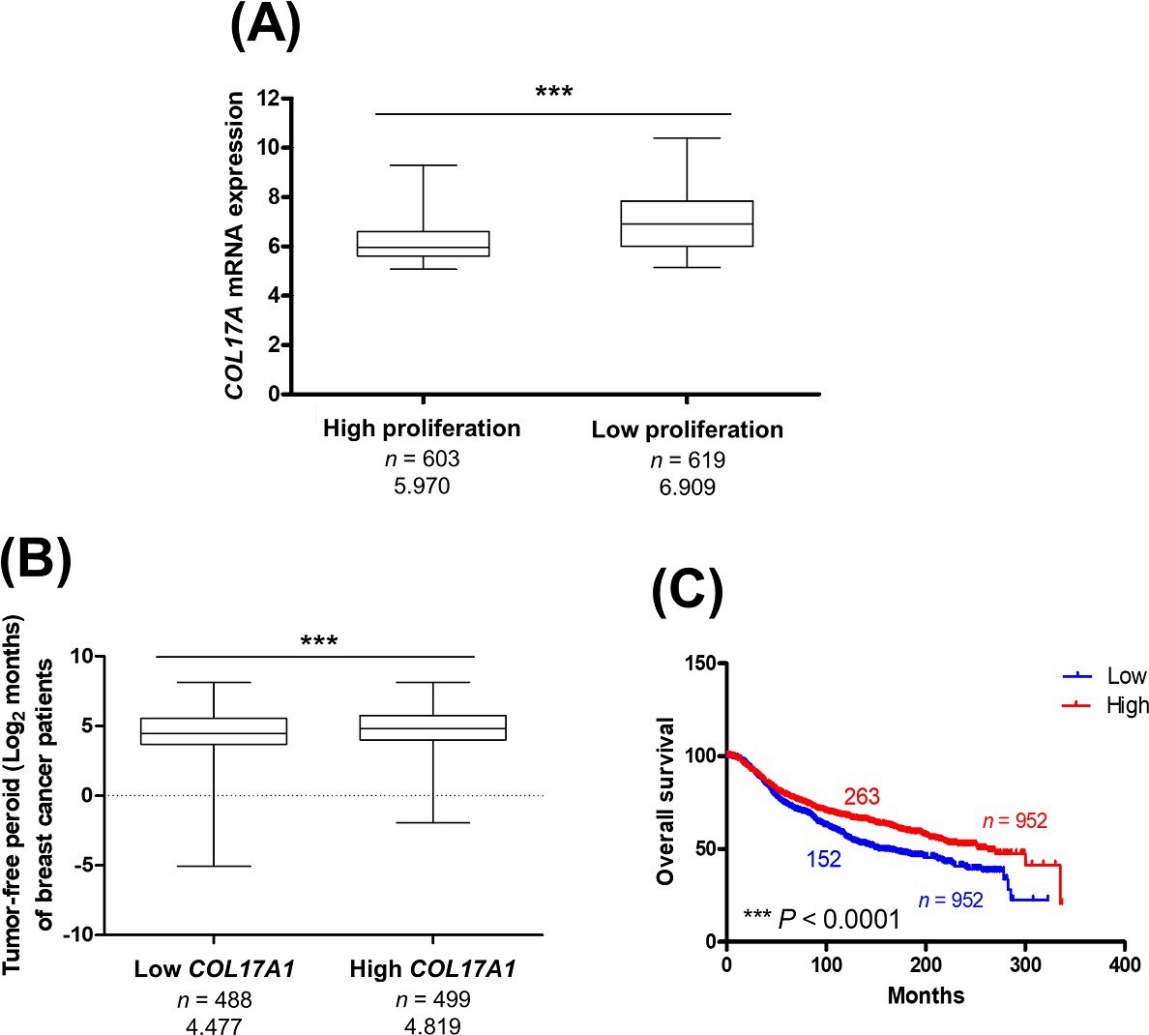
High *COL17A1* level is associated with low proliferation and extended tumor progression in patients. The *COL17A1* mRNA expression in primary breast cancer and patient clinical data were retrieved from METABRIC study. **(A)** Boxplot analysis of *COL17A1* level in low-proliferation and high-proliferation ER+ HER2− tumors categorized by 3-gene classifier subtype. **(B–C)** The *COL17A1* level was categorized as low and high expression comparing to median expression. Tumor-free period **(B)** and overall survival **(C)** of patients bearing tumors with high and low level of *COL17A1* were analyzed. Log-rank test was used for survival analysis. Mann−Whitney *U*-test was used for boxplot. ***P < 0.0001.

The associations of *COL17A1* expression and tumor progression were explored in breast cancer patients using data from the METABRIC study [18]. Fig 7A revealed a significantly high level of *COL17A1* in low-proliferation compared to high-proliferation tumors. In addition, patients with a high level of *COL17A1* demonstrated an extended tumor-free period and overall survival time, as shown in Fig 7B and 7C. To investigate whether *COL17A1* is an independent prognostic factor, multivariate analyses were performed with several clinical variables as covariates, i.e., *p53* status, age, subtype, and staging. Table 1 showed that *COL17A1* level was significantly prognostic of overall survival independently of other clinical factors. We therefore suggest that the inhibitory effects of *COL17A1* on cell growth and proliferation could lead to the prevention of tumor progression and prolonged survival time in breast cancer patients.

**Table 1:** Univariate and multivariate Cox regression model using METABRIC database.

Variable	Reference	Univariate analysis		Multivariate analysis	
		HR (95% CI)	*P*-value	HR (95% CI)	*P*-value
*COL17A1* level (≥ median)	Low level (< median)	0.75 (0.66−0.85)	4.78 × 10^−6^	0.87 (0.76−0.99)	0.0387
*p53* status (mutant)	Wild-type	1.27 (1.11−1.45)	0.0004	1.27 (1.08−1.49)	0.0029
Age ≥ 50	< 50	1.73 (1.46−2.06)	5.37 × 10^−10^	1.68 (1.40−2.01)	1.31 × 10^−8^
Subtype			1.05 × 10^−8^		0.0001
ER+ HER2− High proliferation	ER+ HER2− Low proliferation	1.47 (1.27−1.70)	2.16 × 10^−7^	1.25 (1.07−1.46)	0.0051
HER2+		1.71 (1.39−2.11)	4.40 × 10^−7^	1.46 (1.16−1.84)	0.0013
ER− HER2−		1.14 (0.94−1.38)	0.1909	0.94 (0.75−1.18)	0.6058
Stage			3.96 × 10^−21^		1.10 × 10^−18^
Stage I	Stage 0	0.55 (0.46−0.66)	7.47 × 10^−11^	0.57 (0.48−0.69)	1.26 × 10^−9^
Stage II		0.98 (0.85−1.14)	0.8192	0.99 (0.85−1.15)	0.8710
Stage III		1.75 (1.36−2.25)	1.63 × 10^−5^	1.67 (1.30−2.16)	0.0001
Stage IV		3.38 (1.67−6.82)	0.0007	3.50 (1.73−7.11)	0.0005

HR, hazard ratio; CI, confidence interval; Each variable was analyzed in 760 breast cancer patients.

## Discussion

COL17 has been reported to be as an adhesion molecule and receptor that mediates signaling pathways in skin epithelia [9,19], but its function in other tissues has rarely been studied. The association between COL17 expression and tumor progression has been reported, but it remains controversial in different cancers [15]. Research on the role of COL17 in various types of cancer, including colorectal, lung and breast cancers, has mostly focused on tumor migration, invasion and metastasis [13,16,20]. Regards to an originally low expression of COL17 in breast cancer cell lines [21], we generated the stable inducible COL17-expressing COL cells to study cell proliferation and growth *in vitro* and tumor progression *in vivo*. In the tetracycline-regulated lentiviral expression system, doxycycline binds to the Tet repressor and allows COL cells to express COL17 (S1 Fig). We showed that Dox stimulated COL17 expression in a time-dependent manner, as indicated by the accumulation of the COL17 protein, which increased up to 38-fold following a 14-day treatment period.

The proliferation assay in 2D culture demonstrated that accumulation of COL17 for at least 7 days was sufficient to inhibit the proliferation and viability of the cells. The reduced Ki67 expression proved that COL17 inhibited the cell proliferation in a concentration-dependent manner. Additionally, the colony formation assay showed that overexpression of COL17 influenced the clonogenicity and growth of the cells by reducing the number and size of colonies. The 3D culture results confirmed that COL17 affected the proliferation and growth of cells in spheroids by reducing cell viability and decreased the spheroid size; however, this effect was significant at day 12, which was later than that in the 2D system. It is possible that the distinctions in growth and architecture between 2D- and 3D-cultured MCF7/COL cells are involved in the delayed response of spheroids to COL17.

**Fig 8:**
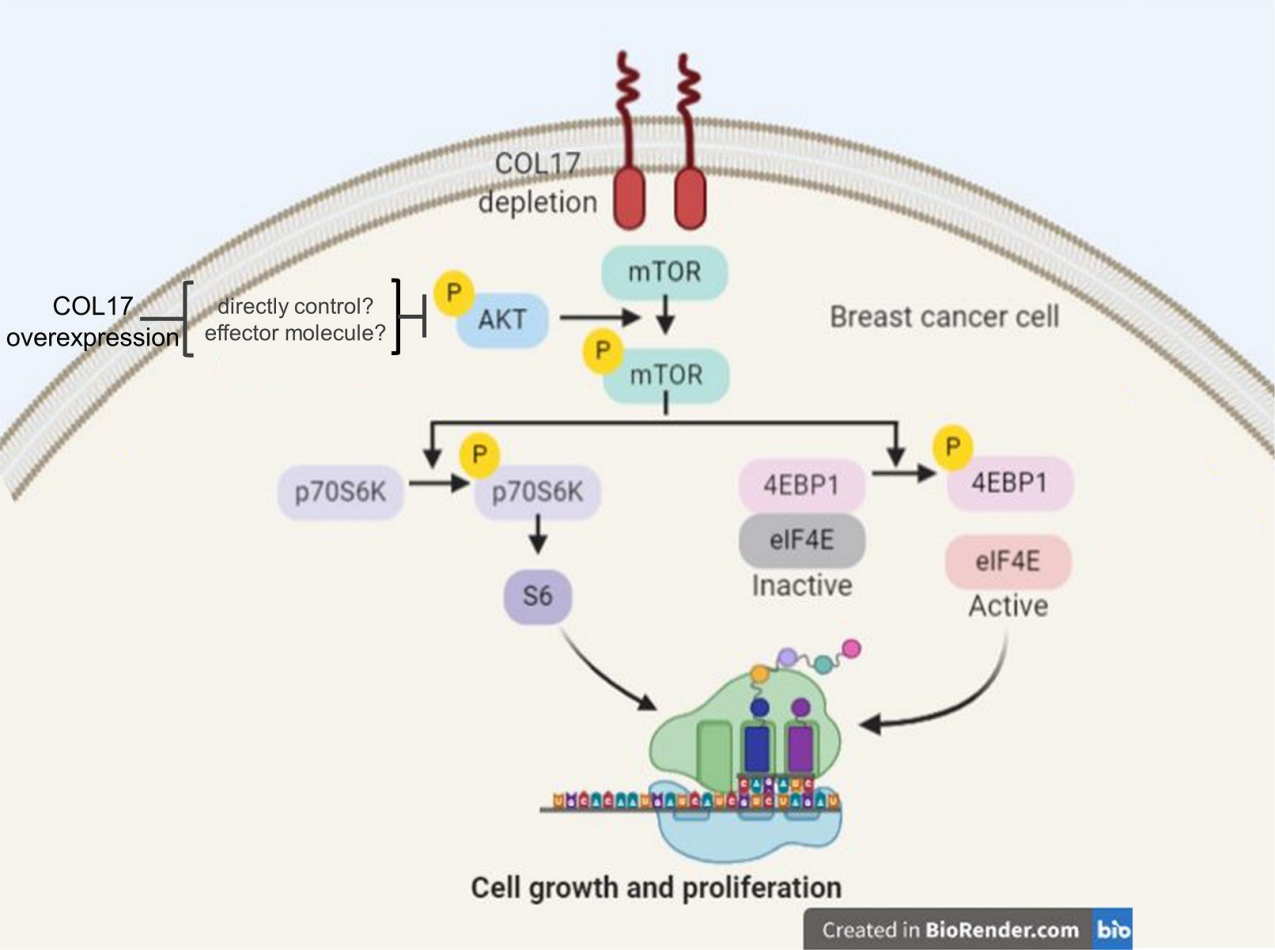
Proposed schematic presentation of the COL17 effect on the AKT/mTOR signaling pathway. The AKT/mTOR signaling pathway promotes cell proliferation and growth by phosphorylation of its effector proteins. The phosphorylated p70S6K subsequently phosphorylates S6 ribosomal protein to induce ribosome synthesis. During inactive state, eIF4E binds to its inhibitory partner 4EBP1. The phosphorylated 4EBP1 releases the binding to eIF4E making it active and promote the protein translation. In breast cancer tissues, COL17 has been found to be depleted. The overexpression of COL17 deactivates the AKT/mTOR pathway by dephosphorylation of AKT, mTOR, p70S6K, and 4EBP1 leading to inhibition of cell growth and proliferation. The binding partners and effector molecules of COL17 which directly control the AKT/mTOR pathway in breast cancer are unknown and need to be further clarified.

A recent study showed that the overexpression of COL17 suppresses skin hypertrophy in aged skin and that loss of COL17 results in skin hypertrophy in neonatal mice [22]. The role of COL17 in the proliferation of the interfollicular epidermis is mediated through the Wnt/β-catenin signaling pathway [22]. In this study, we clarify that COL17 overexpression suppresses the cell proliferation and growth of breast cancer and attenuates the AKT/mTOR signaling molecules (Fig 8). AKT/mTOR signaling molecules can be initially activated by a range of signals leading to interactions with their receptors, including hormones, growth factors, and components of the extracellular matrix [23]. Activation of this pathway promotes tumor growth and plays a significant role in endocrine resistance in breast cancer [24]. Consistent with our result, cell migration and proliferation of keratinocytes are suppressed by the COL17 through the downregulation of PI3K/AKT/mTOR pathway; and this is mediated via α6β4 integrin [25,26]. Conversely, the absence of COL17 activates this pathway via β4 integrin and the focal adhesion kinase (FAK) resulting in Rac1 activation and reduced cell migration [26]. These previous studies demonstrated the effects of COL17 on several molecules involved with upstream and downstream of AKT/mTOR pathway in keratinocytes, which should also be explored in other cell types.

AKT and its downstream target mTOR are protein kinases that are activated by phosphorylation of two target amino acids, serine (Ser) and threonine (Thr) [24,27,28]. Activated AKT and mTOR play important diverse roles in metabolic regulation, cell proliferation and regulation of cell death [29]. Our study demonstrated that 2- and 7-day induction of COL17 expression inhibited phosphorylation at Ser473 of AKT (Fig 5A); this was reported to be associated with ubiquitination of AKT, resulting in AKT degradation [30]. The reactivation of AKT was observed on day 14, suggesting compensatory regulation related to conservation of AKT levels that is essential for cell survival. However, phosphorylated mTOR expression was markedly suppressed by COL17 for 14 days (Fig 5B). These findings suggested that the COL17 expression level might be a predictive biomarker for unresponsiveness to mTOR/AKT inhibitors, called rapalogs, such as everolimus that has been approved for breast cancer treatment [31]. Whether mTOR is a major regulator of COL17-mediated breast cancer cell growth needs to be clarified using mTOR inhibitor-treated cell [26]. Further research should focus on how COL17 affects the AKT/mTOR pathway (Fig 8), which could be regulated through integrin α6β4, a direct binding partner of COL17, as shown in keratinocytes [26].

mTOR promotes cell growth and proliferation by adding a phosphoryl group to two effectors, p70S6K and 4EBP1 [24] (Fig 8). After phosphorylation, pp70S6K subsequently phosphorylates S6 ribosomal protein to induce ribosome synthesis [32]. Moreover, the eukaryotic translation initiation factor 4F complex, consisting of eIF4E, eIF4A and eIF4G, recruits 40S ribosomal subunits to the 5′ end of mRNA to initiate protein synthesis [33]. During the inactive state, eIF4E binds to its inhibitory partner, hypophosphorylated 4EBP1. Phosphorylated 4EBP1 releases eIF4E, making it active, and promoting protein translation [34]. This study revealed that COL17 significantly inhibited the phosphorylation of 4EBP1 for 14 days (Fig 5D), exhibiting maximum inhibition at 7 days, which is similar to the inhibition pattern of mTOR (Fig 5B). Recovery of p4EBP1, detected at 14-day induction of COL17, has been reported within 6 hours of initial inhibition by rapamycin, an mTOR inhibitor [35]. Previous studies have shown that 4EBP1 phosphorylation is sensitive to and reversible by several stimuli, including cytokines, drugs, growth factors, and hormones [34,36]. In addition, 4EBP1 phosphorylation can be activated by several protein kinases in other pathways, including GSK3β, MAPK, ERK and CDK1 [37–40]. In breast cancer, several signaling pathways are involved in cancer development and progression, including the estrogen, HER2, Wnt/β-catenin, CDK and breast cancer kinase pathways [41]. Thus, whether COL17 regulates cell growth and proliferation through other pathways could be further investigated.

To confirm the effect of COL17 on tumor progression and survival time *in vivo*, stable MDA-MB-231/COL cells were used for xenograft. Mice implanted with COL17-expressing cells showed increased tumor-free survival, decreased primary tumor size, and extended overall survival time. The patient data indicated that high *COL17A1* expression was associated with low-proliferation tumors, increased tumor-free and overall survival times. In Addition, the *COL17A1* level is an independent prognostic factor for better survival of breast cancer patients. These results support the crucial role of COL17 in the development and progression of breast cancer.

In conclusion, we revealed the novel function of COL17 in inhibiting the growth and proliferation of breast cancer cells. Increased expression of COL17 in breast cancer cells reduces the expression of the proliferative marker Ki67 and deactivates mTOR signaling molecules by dephosphorylation of AKT, mTOR, and effector molecules, including p70S6K and particularly 4EBP1. We provide evidence that high expression of COL17 at the protein and gene levels is related to low proliferation, increased tumor-free and overall survival times. These results suggest the potential role of COL17 as a novel target for breast cancer treatment.

## Materials and methods

### Cell lines and treatments

The original cell lines, MCF7 (ATCC HTB-22^TM^) and MDA-MB-231 (ATCC HTB-26^TM^), were purchased from the American Type Culture Collection. The stable cell lines, COL17-overexpressed MCF7 and MDA-MB-231 cells, were generated using the tetracycline-regulated lentiviral expression system (S1 Fig). In brief, the full-length *COL17A1* genomic DNA fragment was cloned into the entry vector (pENTR™3C), integrated into the destination vector (pLenti6.3/TO/V5- DEST) using Gateway LR Clonase II enzyme mix, and then transformed into Stbl3 cells to generate the expression clone. The *TetR* construct was co-transduced at 10 MOI into the cells with either the *COL17A1* construct for COL17-expressing cells (COL cells) or empty vector for controls (mock cells). The transduced cells were selected using Blasticidin and Geneticin^®^ for 3 weeks. After 3 weeks, the cells were cultured in complete media without the selection antibiotics. MCF7 cells were cultured with the Minimum Essential Medium Eagle (MEM) containing 10% fetal bovine serum (Gibco^TM^, Thermo Fisher Scientific), 1mM of sodium pyruvate and 0.1mM of MEM non-essential amino acid, 100 U/ml penicillin, and 100g/ml streptomycin. MDA-MB-231 cells were cultured with the Dulbecco’s Modified Eagle’s medium (DMEM) containing 10% fetal bovine serum, 100 U/ml penicillin, and 100g/ml streptomycin. Cells were incubated in 5% CO_2_ at 37°C. To induce the expression of COL17, cells were treated with doxycycline hydrochloride (Dox; Sigma-Aldrich Merck) at 1 μg/ml in the corresponding complete media and the media were refreshed every 2 days.

### Two-dimensional (2D) proliferation assay

The stable MCF7/COL and mock cells were seeded (1,500 cells/well) in 96-well plates. At 48 hours after seeding, the cells were treated with Dox and continuously cultured up to 10 days of treatment. Cells were duplicately counted with hemocytometer (*n* = 5 per group) to evaluate the total cell number at 0, 3, 5, 7, and 10 days of Dox treatment. An MTT (3-(4,5-dimethylthiazol-2-yl)-2,5-diphenyltetrazolium bromide) assay was used to evaluate the cell viability. The MTT assay was performed by incubating cells with 0.05 mg/ml of MTT in serum-free MEM at 37°C in 5% CO_2_ for 3 hours (*n* = 5 per group). The reagent was then removed, replaced with dimethyl sulfoxide to dissolve formazan crystal, and further incubated for 1 hour. The absorbance of the reaction was measured at the wavelength of 562 nm using a microplate reader (CLARIOstar^®^, BMG Labtech).

### Analysis of cell death

The stable MCF7/COL and mock cells were seeded (1,500 cells/well) in 96-well plates. At 48 hours after seeding, the cells were treated with Dox and continuously cultured up to 10 days of treatment. At 10 days of Dox treatment, nuclear staining with Hoechst 33342 was performed to analyze the cell death. The staining was performed by incubating the cells with 1 μg/ml of Hoechst 33342 (Sigma, MO, USA) in culture media at 37°C in 5% CO_2_ for 15 minutes in the dark (3 areas per sample and 3 samples per group). The stained nuclei were visualized under fluorescence microscope. Cells were counted by ImageJ program. The dead cells were identified following the Cold Spring Harbor Protocols [42]. The number of dead cells was normalized to the total cell number and reported as percentage of cell death.

### Three-dimensional (3D) proliferation assay

The stable COL17-overexpressed MCF7 cells were seeded (1,500 cells/well) into 96-ultra-low attachment plates (PerkinsElmer). At 48 hours after plating, cells were treated with Dox and continuously cultured up to 12 days of treatment. The images of spheroids were recorded using a phase-contrast microscope with 10× magnification. The spheroid area was measured using an image processing program (ImageJ program, NIH), excluding the intra-spheroid spaces, normalized with the control, and reported as relative spheroid size (*n* = 5 per group). Cell viability of spheroid was evaluated by an ATP assay using ATPlite™ 3D ATP kits (PerkinsElmer) according to the manufacturer’s instruction. Briefly, spheroids were incubated with 100 μl of a lysis buffer for 10 minutes and then 100 μl of substrate were added. The relative luminescent unit (RLU) of each sample was measured with a luminometer (CLARIOstar^®^, BMG Labtech). The RLU of the Dox-treated cells was normalized with that of the untreated cells (*n* = 5 per group).

### Colony formation assay

The stable COL17-overexpressed MCF7 cells were seeded (500 cells/well) into 6-well plates. At 48 hours after plating, cells were treated with Dox and continuously cultured up to 12 days of treatment. Cells were fixed with methanol for 1 hour and incubated overnight with 0.1% crystal violet in phosphate saline buffer. After staining, images of cell colonies were analyzed by ImageJ program. The number of colonies, total area, and colony sizes of the Dox-treated group were normalized with those of the untreated group and reported as a ratio (*n* = 3 per group).

### Antibodies

For western blotting, anti-collagen XVII (ab184996) and HRP-conjugated secondary antibody (ab205718) were purchased from Abcam, MA, USA. The anti-pAKT (9271), anti-AKT (9272), anti-pmTOR (5536), anti-mTOR (2983), anti-pp70S6K (9234), anti-p4EBP1 (2855), and anti-β-actin (4970) antibodies were purchased from Cell Signaling Technology, MA, USA. The anti-Ki67 (ab16667, Abcam) and HRP-conjugated secondary antibodies (K4002, Dako, Japan) were used for immunocytochemistry.

### Western blot analysis

Cells were lysed using the chilled radio-immunoprecipitation assay (RIPA) buffer, containing 150 mM NaCl, 1.0% IGEPAL^®^ CA-630 (Sigma-Aldrich), 0.5% sodium deoxycholate, 0.1% sodium dodecyl sulfate (SDS), 50 mM tris(hydroxymethyl) aminomethane pH 8.0 (Sigma Aldrich) and a cocktail protease inhibitor (Sigma Aldrich). The cell lysate was sonicated and centrifugated at 15,000 rpm for 15 minutes at 4°C. The supernatant was subjected to protein concentration measurement using the BCA^TM^ protein assay (Thermo Fisher Scientific). The protein pellet was resuspended in a Laemmli buffer containing β-mercaptoethanol (Biorad) and boiled for 2 minutes prior to protein determination by western blotting. From each cell lysate, 30 μg of protein was separated by 7.5% sodium dodecyl sulphate-polyacrylamide gel electrophoresis (SDS-PAGE) and transferred to a PVDF membrane (Immobilon^®^, Merck), which was subsequently blocked in 5% non-fat dried milk (Biorad). Immunoblotting was performed by incubating the membrane with primary antibody for overnight and further incubated with horse-radish peroxidase (HRP)-conjugated secondary antibodies for 1 hour at room temperature. Dilutions of antibodies were used according to the manufacturer’s instruction. The immunoreactive bands were developed by an ECL substrate (Santa-Cruz) and visualized using a chemiluminescent luminescent image analyzer (LAS-4000 mini, Fujifilm, GE Healthcare). The expression level of target proteins (*n* = 3 per group) was analyzed by an ImageJ software. Protein extraction from media was performed as described in the previous study [16]. In brief, the media were replaced with an antibiotic-free media for 12 hours prior to the extraction. Proteins were precipitated by incubating chilled acetone for 1 hour at −80°C and subsequently centrifuged at 13,000*g* for 15 minutes at 4°C. The precipitated protein was resuspended in Laemmli buffer containing β-mercaptoethanol (Biorad) and boiled for 5 minutes before loading to SDS-PAGE. The blotting method of media protein was similar to those of the cell lysate.

### Immunocytochemistry of Ki67

The stable MCF7/COL cells were seeded (8,000 cells/well) on poly-L-lysine coated glass coverslips in 24-well plates, treated with Dox, and continuously cultured up to 12 days of treatment. For immunocytochemistry, cells were fixed with 4% paraformaldehyde for 10 minutes. The samples were incubated with a peroxidase block solution (S2023, Dako) for 15 minutes and subsequently incubated with a protein block reagent (X0909, Dako) for 30 minutes. The primary anti-Ki67 antibody was added and incubated overnight at 4°C. Negative controls were performed by incubating the samples in the absence of the primary antibody. Subsequently, the samples were incubated with the HRP-conjugated secondary antibody. Finally, a DAB chromogen and substrate (K3468, Dako) was added to develop the color of the positive area. Immunoreactivity was visualized under a phase contrast microscope (Nikon Ts2) with 10× magnification. The quantitative expression of Ki-67 was calculated by ImageJ software using the percentage of Ki-67 positive area normalized by the total number of cells. The result was displayed as a ratio of the normalized Ki-67 expression between the treated cells and the untreated cells. The experiment was performed by 3 independent areas per sample with 3 samples per group.

### Ethics statement

All experimental protocols were approved by the Faculty of Pharmaceutical Sciences, Chulalongkorn University Animal Care and Use Committee, Bangkok, Thailand (Number 1933008). All procedures were done in accordance with the relevant guidelines and regulations. Research staffs had attended the training by the Institute of Animals for Scientific Purpose Development, Thailand.

### Animal experiments

The BALB/c nude female mice were purchased from Nomura Siam^®^ (Nomura Siam International, Nakorn Pathom, Thailand). All mice were taken care under strictly hygienic conventional plastic cages at 25°C; 12–12 light dark cycle. Food and water were supplied *ad libitum*. Corn cub was used as bedding media in each cage and was refreshed every week. Two mice were kept in each cage, size 16.5 × 27 × 13 cm^3^ (W × L × H). Mouse shelter was used as enrichment for each cage. Eight mice were allocated into two groups, implanted with MDA-MB-231/COL (MDA/COL) cells or mock cells (*n* = 4 per group). At 48 hours before grafting, the stable MDA /COL and mock cells were treated with Dox 1 μg/ml in the complete media. The water containing Dox at 2 mg/ml in 1% sucrose was supplied for both groups of mice from 4 days before grafting and continuing throughout the experiment. Xenograft was performed at 7 days after receiving the mice. Mice were anesthetized with 60 mg/kg pentobarbital sodium by intraperitoneal injection and were observed for the effect of anesthesia by toe pinching and respiratory rate observation. Each 6-week-old mouse was grafted for 2-injection sites at right and left inguinal mammary fat pads with 2 × 10^6^ pretreated cells per site. After the surgery, all mice were received 5 mg/kg of carprofen (Rimadyl^®^) as an analgesia. After surgery, mice were place on sanitary pad and electric hot blanket, which strictly control the heat to avoid skin burnout. Mice were transferred to the cage when completely recovered. The COL17 expression was induced by treating the mice with 1mg/ml Dox in drinking water. The water was replaced every 2 days. To evaluate tumor volume, the greatest longitudinal length and the width of tumor were measured using caliper. The calculation of tumor volume was based on modified ellipsoidal formula [43,44] as follows: *Tumor volume = 1/2(length × width*^*2*^*)”*. The non-parametric Mann−Whitney *U*-test was used to compare the difference of tumor volume between two groups.

Mice were monitored for behavior, bodyweight, and tumor size using a vernier caliper every 2 days up to 9-week post-xenograft. The monitored behaviors include level of consciousness (alert/depress), grooming, and feeding pattern. Pain and distress were assessed by these indicators: inactivity, loss of appetite, loss of mobility, labor breathing, restlessness, weight loss (≥ 20%) and guarding the painful area. If mice demonstrated signs of pain, analgesia would be applied. However, pain and distress were not detected in any mice throughout the experiment. The humane endpoint was used following these criteria: tumor size diameter ≥ 20 mm; bodyweight loss ≥ 20%; and the presence of ulceration on the tumor. Within 24 hours after the humane endpoint, mice were sacrificed using intraperitoneal injection of overdose sodium pentobarbital at 100 mg/kg. None of the mice died before meeting criteria for euthanasia. The confirmation of euthanasia was performed by inducing pneumothorax and removing vital organs. The tumor cells from the primary implantation site were harvested for total protein determination by western blot analysis.

### METABRIC analysis

The *COL17A1* mRNA expression in primary breast cancer and patient clinical data from METABRIC study [18] were retrieved from cBioPortal [45]. Boxplot analysis was used to compare *COL17A1* level between low-proliferation and high-proliferation ER+ Her2− tumors categorized by 3-gene classifier subtype [46]. The level of *COL17A1* expression was categorized as low and high expression comparing to median *COL17A1* expression for subsequent analysis of tumor-free period and overall survival. Patients with unknown data of *COL17A1* expression, time to tumor-free and overall survival were excluded. Cox regression model was used for univariate and multivariate analyses of the following variables: *COL17A1* expression level, *p53* status, patient age, tumor subtypes by 3-gene classifier, and pathological stage (0–IV).

### Statistical analysis

All data were reported as mean ± standard deviation. Statistical analysis was done by GraphPad Prism 5.0, GraphPad Software, San Diego, California, USA. The difference between two groups was analyzed by two-tailed Student’s *t*-test. The Welch’s adjusted *t*-test was used if an unequal variance between groups was observed. Mann−Whitney *U*-test was used for patient and mouse data. Significant difference was indicated at a 95% confidence interval (*P* < 0.05). Median overall survival data were demonstrated as a Kaplan-Meier survival curve and the difference was compared using the logrank (Mantel-Cox) test.

## Supporting information

S1 Fig Tetracycline-regulated lentiviral expression system. (PDF)

S2 Fig Proliferation of MCF7/COL and mock cells in 2D culture. (PDF)

S3 Fig Analysis of cell death. (PDF)

S4 Fig Mammary fad pad xenograft. (PDF)

S1 Raw images (PDF)
