# Supplementary material for: Collagen XVII inhibits breast cancer cell proliferation and growth through deactivation of the AKT/mTOR signaling pathway
Source: PLoS One. 2021 Jul 22;16(7):e0255179. doi: 10.1371/journal.pone.0255179 (PMC8297889; doi:10.1371/journal.pone.0255179)

S1 Fig. Tetracycline-regulated lentiviral expression system.

(A) Construction of stable cell lines.
(1) The full-length *COL17A1* genomic DNA fragment was cloned into the entry vector (pENTR™3C) using forward primer: 5’-GCCGCCATGGATGTAACCAAGAAAAACAAAC-3’ and reverse primer: 5’-TCACGGCTTGACAGCAATACTTC-3’. The *COL17A1* fragment was integrated into the destination vector containing Blasticidin resistant gene (pLenti6.3/TO/V5-DEST) using Gateway LR Clonase II enzyme mix and then transformed into Stbl3 cells to generate the expression clone. **(2)** Lentiviruses containing *COL17A1* expression construct, empty vector, or Tet repressor (*TetR*) vector with Neomycin resistant gene (pLenti3.3/TR) were produced in 293FT cells using ViraPower™ Packaging Mix according to the manufacturer’s protocol. **(3)** The virus-containing media were harvested and used to determine the viral titer by qPCR. **(4)** The *TetR* construct was co-transduced at 10 MOI into MCF7 and MDA-MB-231 cells with either the *COL17A1* construct for COL17-expressing cells or empty vector for mock cells. **(5)** The transduced cells were selected and continuously cultured in media containing Blasticidin and Geneticin® for 3 weeks. **(B)** In the absence of doxycycline (Dox), TetR binds to Tet operator and suppresses the transcription. **(C)** In the presence of Dox, Dox binds to the TetR and allows the COL17A1 (COL) cells to express COL17.

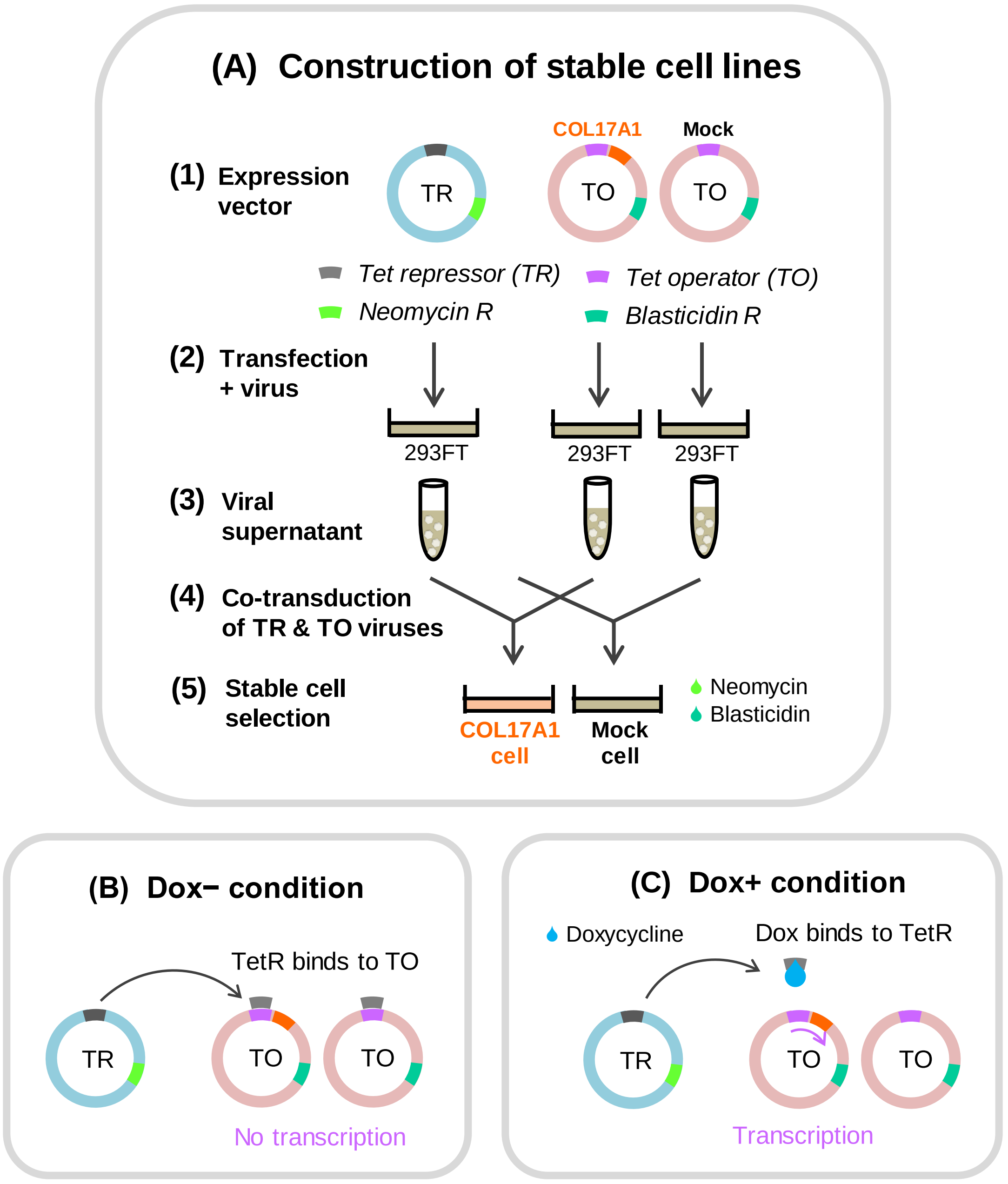

Supplement: S1 Fig — (PDF) [file pone.0255179.s001.pdf]
