# Supplementary material for: Collagen XVII inhibits breast cancer cell proliferation and growth through deactivation of the AKT/mTOR signaling pathway
Source: PLoS One. 2021 Jul 22;16(7):e0255179. doi: 10.1371/journal.pone.0255179 (PMC8297889; doi:10.1371/journal.pone.0255179)
Supplement: S2 Fig — (PDF) [file pone.0255179.s002.pdf]

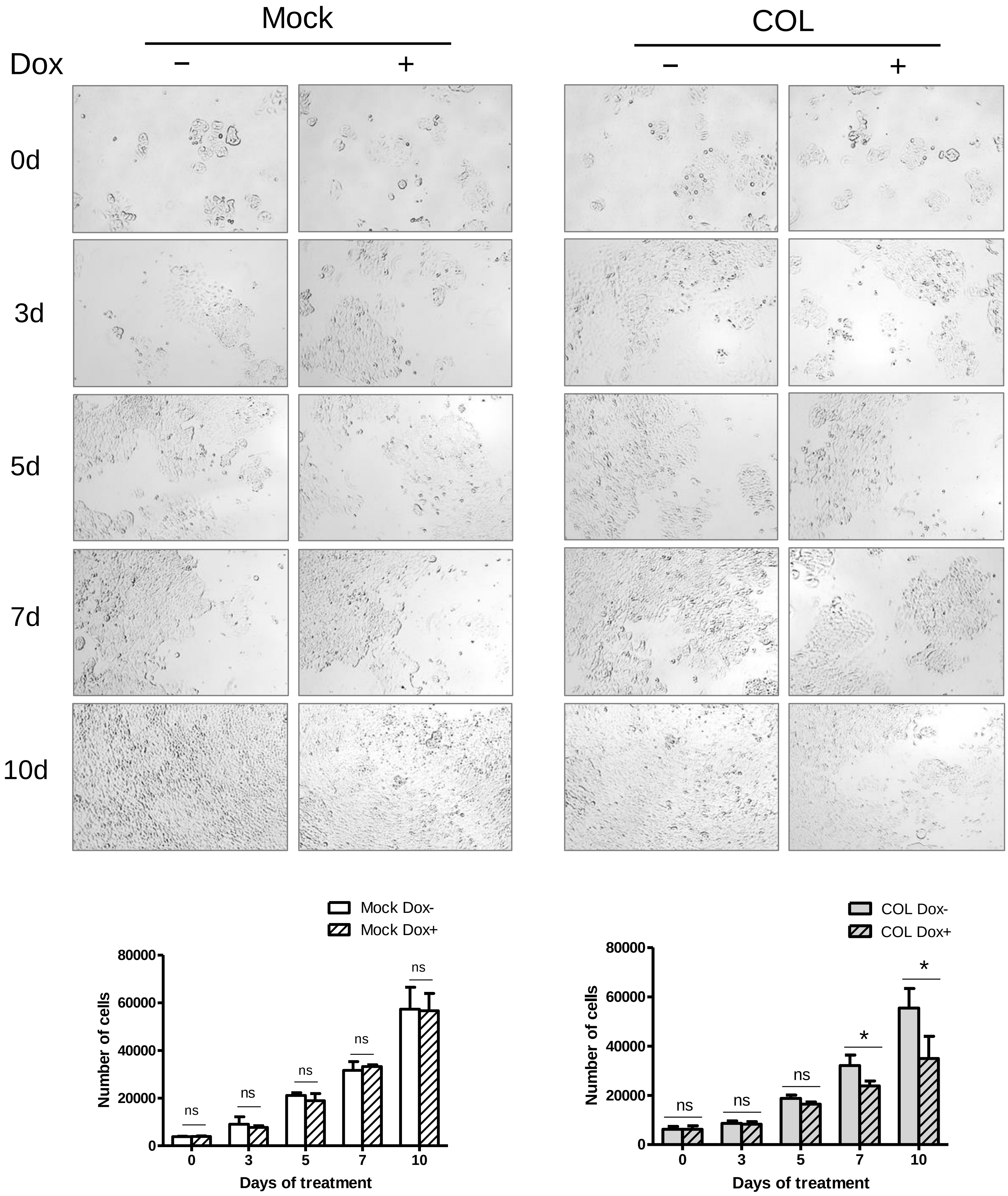

S2 Fig. Proliferation of MCF7/COL and mock cells in 2D culture.

MCF7/COL (COL) and mock cells were grown in 96-well plate for 48 hours and then treated with 0.1 μg/ml of doxycycline (Dox+) or not treated (Dox−). The represented images of cell culture were taken before and at 3, 5, 7 and 10 days of Dox treatment. The number of cells were determined by cell counting (n = 5 per group). The results of Dox+ were normalized to those of Dox− groups and represented as ratio in Figure 2A. Two-tailed Student's t-test; *$P < 0.05$, **$P < 0.01$, ***$P < 0.0001$; ns, $P \geq 0.05$.
