# Supplementary material for: Collagen XVII inhibits breast cancer cell proliferation and growth through deactivation of the AKT/mTOR signaling pathway
Source: PLoS One. 2021 Jul 22;16(7):e0255179. doi: 10.1371/journal.pone.0255179 (PMC8297889; doi:10.1371/journal.pone.0255179)
Supplement: S3 Fig — (PDF) [file pone.0255179.s003.pdf]

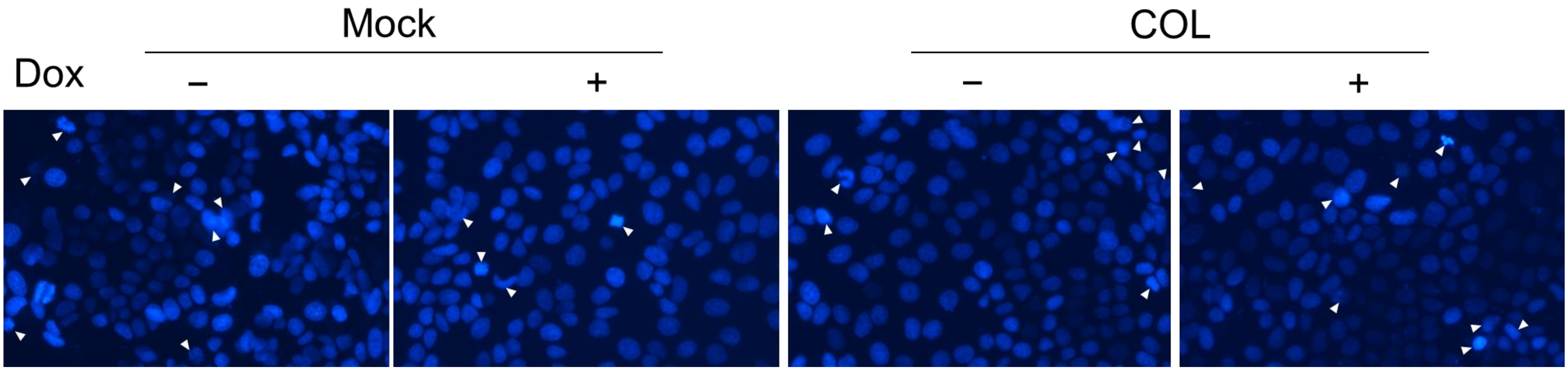

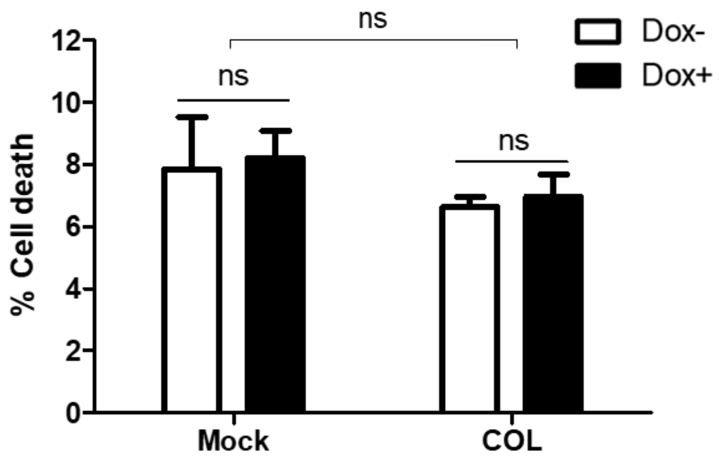

S3 Fig. Analysis of cell death.

The stable MCF7/COL and mock cells were seeded and cultured in 96-well plates with the same condition as used in the 2D proliferation assay. Cells were treated with doxycycline (Dox) at 48 hours after seeding and continuously cultured up to 10 days of treatment. At 10 days of Dox treatment, nuclear staining with Hoechst 33342 was performed to analyze the cell death. The staining was performed by incubating the cells with 1 μg/ml of Hoechst 33342 in culture media at 37° C in 5% CO_2 for 15 minutes in the dark (3 areas/samples and 3 samples per group). The stained nuclei were visualized under fluorescence microscope and the images were recorded with 20× magnification. The dead cells were indicated by arrows. Cells were counted by an image processing program (ImageJ program, NIH). The number of dead cells was normalized to the total cell number and reported as percentage of cell death.
