# Supplementary material for: Collagen XVII inhibits breast cancer cell proliferation and growth through deactivation of the AKT/mTOR signaling pathway
Source: PLoS One. 2021 Jul 22;16(7):e0255179. doi: 10.1371/journal.pone.0255179 (PMC8297889; doi:10.1371/journal.pone.0255179)
Supplement: S4 Fig — (PDF) [file pone.0255179.s004.pdf]

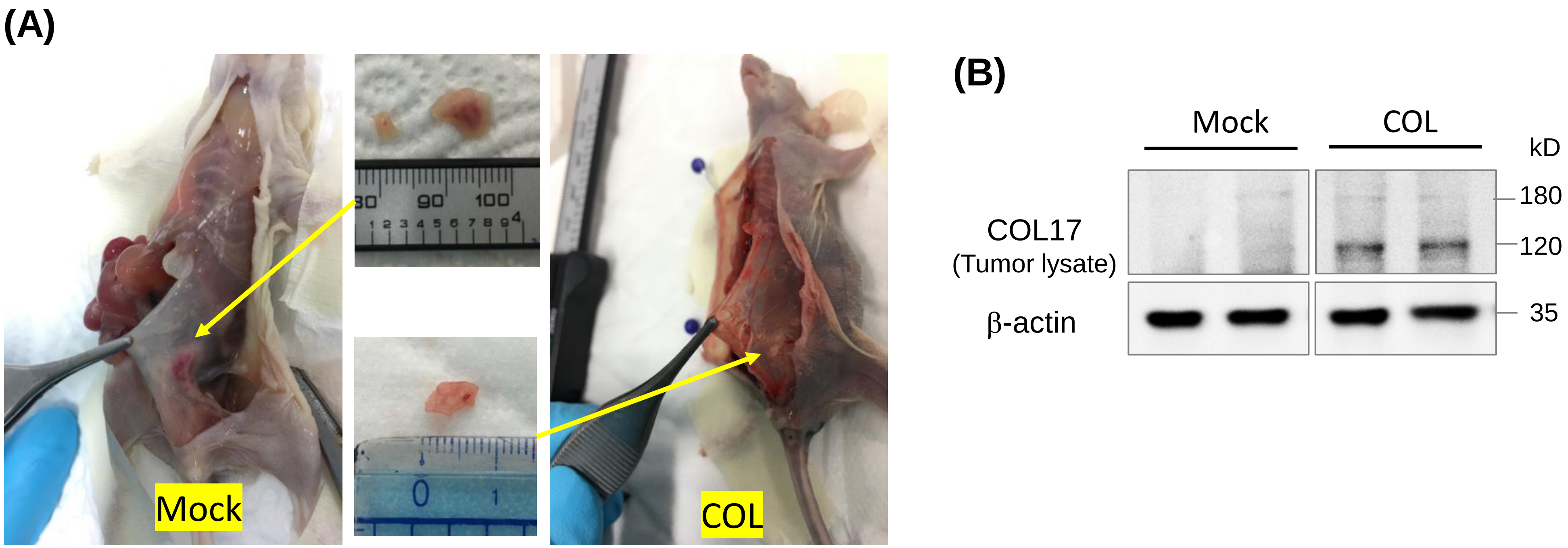

S4 Fig. Mammary fad pad xenograft. The BALB/c nude female mice were implanted with MDA-MB-231/COL (COL) or mock cells at the right and left inguinal mammary fat pads. **(A)** The primary tumors were subjected for total protein extraction and western blot analysis. Arrows indicate the location of primary tumor at the site of implantation. **(B)** Western blot analysis of protein lysates from primary tumors blotting with anti-collagen XVII and anti-β-actin.
