# Supplementary material for: Collagen XVII inhibits breast cancer cell proliferation and growth through deactivation of the AKT/mTOR signaling pathway
Source: PLoS One. 2021 Jul 22;16(7):e0255179. doi: 10.1371/journal.pone.0255179 (PMC8297889; doi:10.1371/journal.pone.0255179)
Supplement: S1 Raw images — (PDF) [file pone.0255179.s005.pdf]

S1_raw_images

Fig 1A

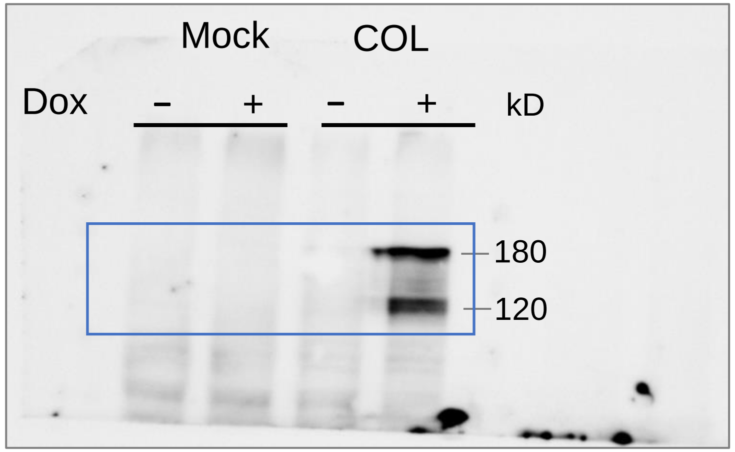

Fig 1A: COL17

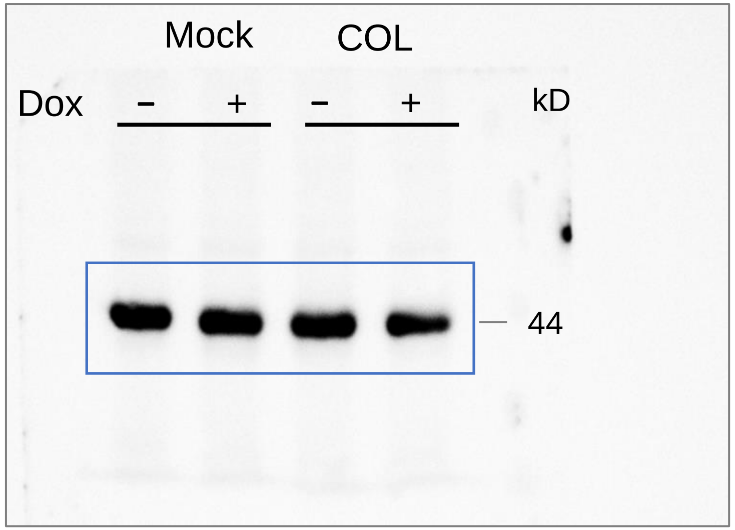

Fig 1A: β-actin

S1_raw_images

Fig 1B

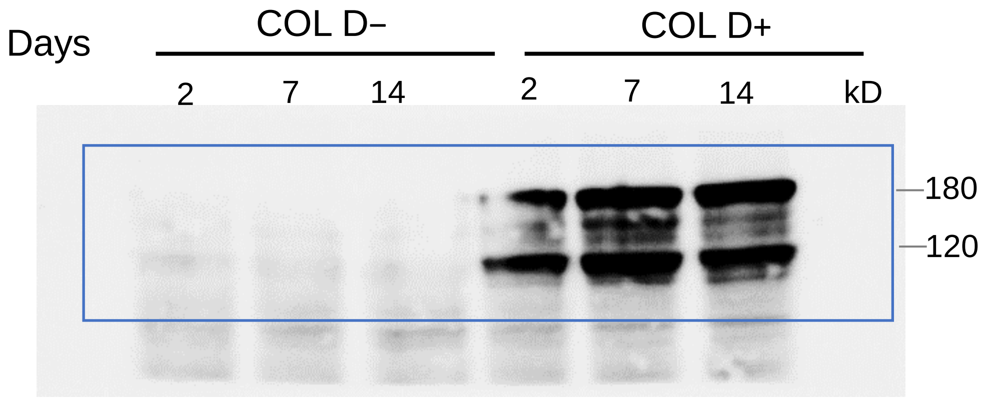

Fig 1B: COL17 (cell lysate)

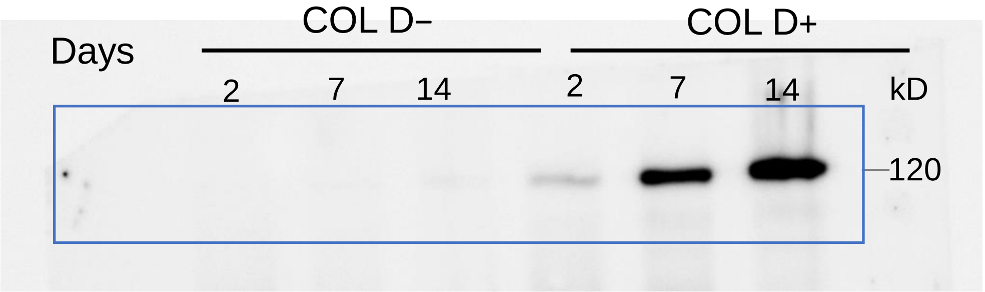

Fig 1B: COL17 (Media)

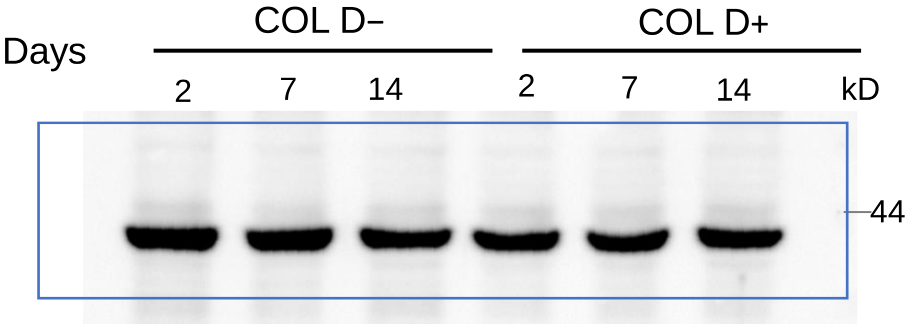

Fig 1B: β-actin

S1_raw_images

Fig 5A

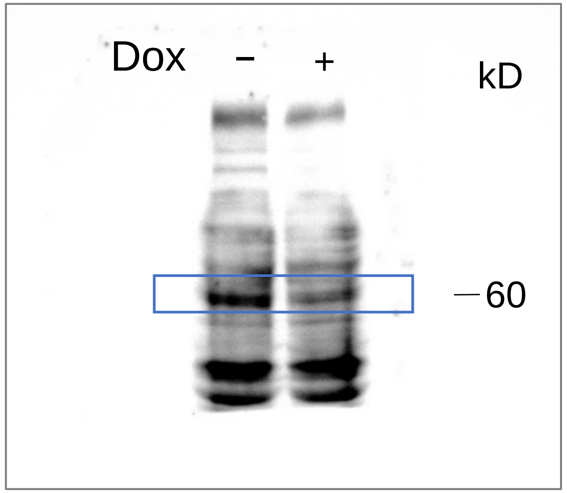

Fig 5A: pAKT 2d

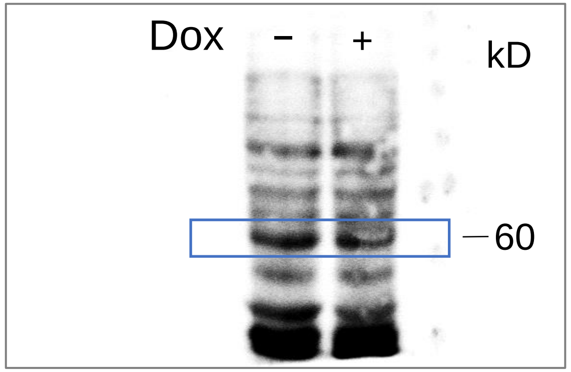

Fig 5A: pAKT 7d

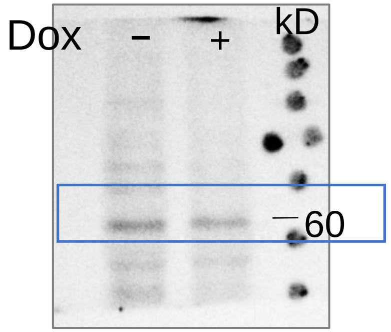

Fig 5A: pAKT 14d

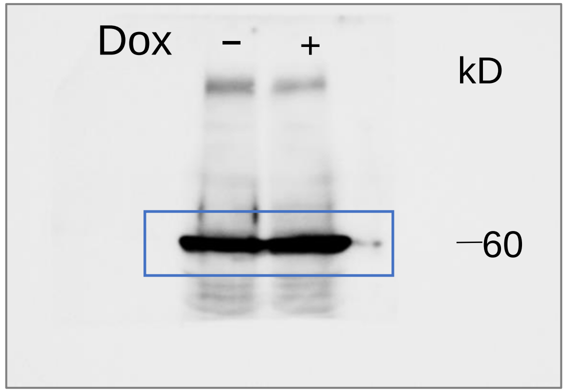

Fig 5A: Total AKT 2d

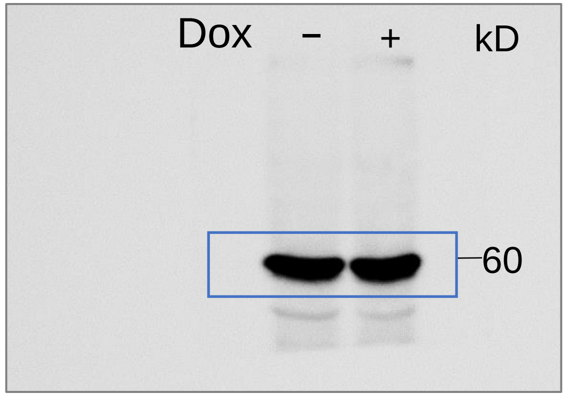

Fig 5A: Total AKT 7d

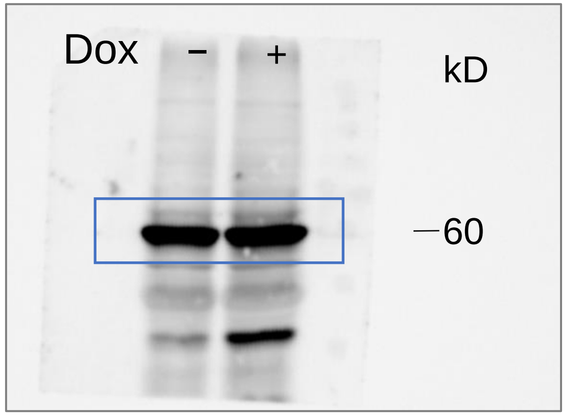

Fig 5A: Total AKT 14d

S1_raw_images

Fig 5B

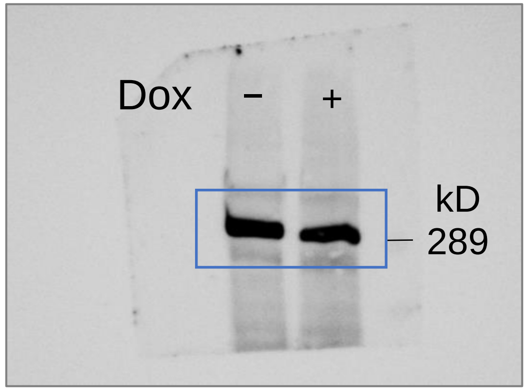

Fig 5B: pmTOR 2d

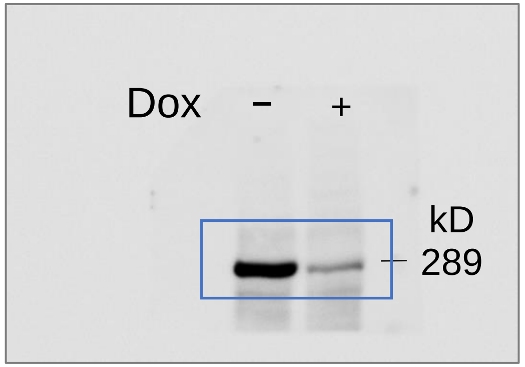

Fig 5B: pmTOR 7d

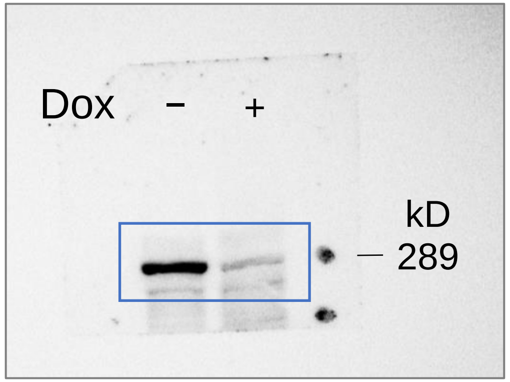

Fig 5B: pmTOR 14d

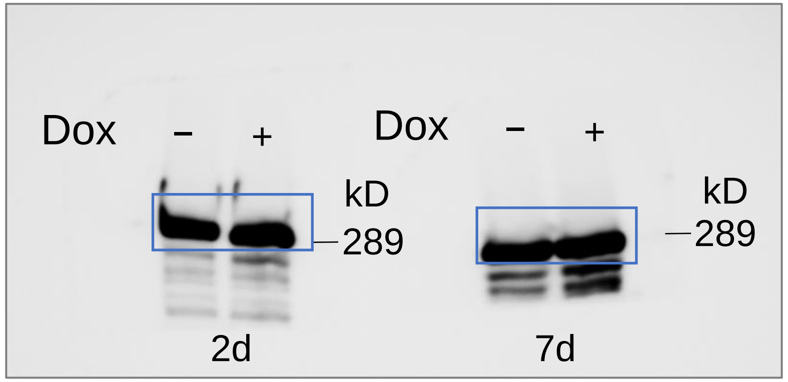

Fig 5B:
Total mTOR
2 and 7d

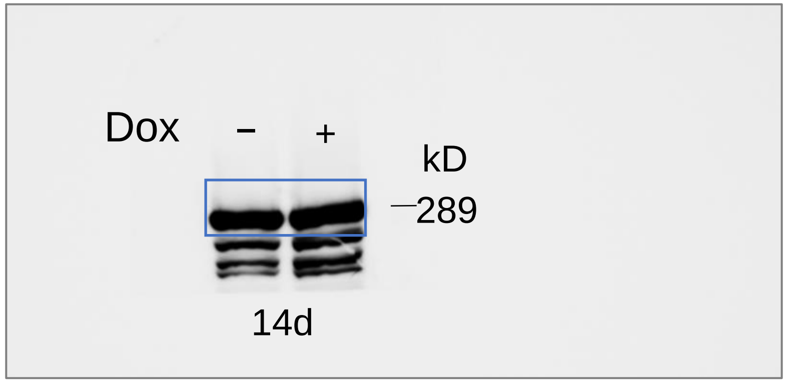

Fig 5B:
Total mTOR
14d

S1_raw_images

Fig 5C

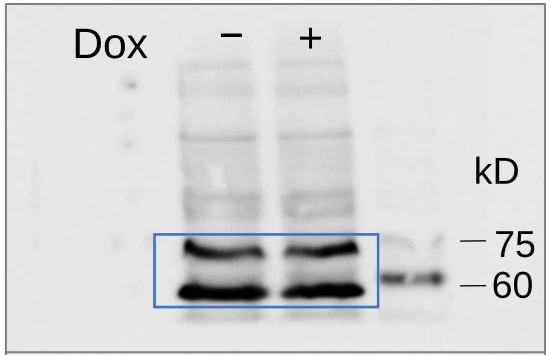

Fig 5C: pp70S6K 2d

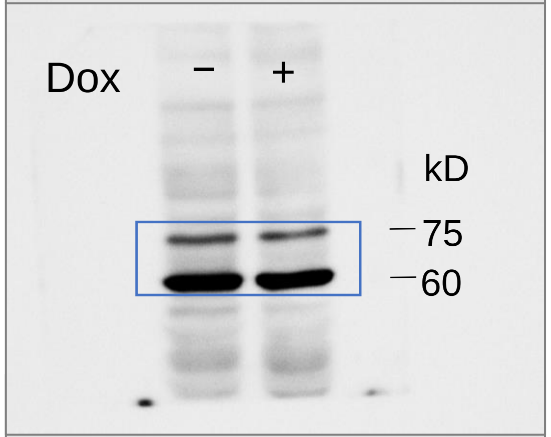

Fig 5C: pp70S6K 7d

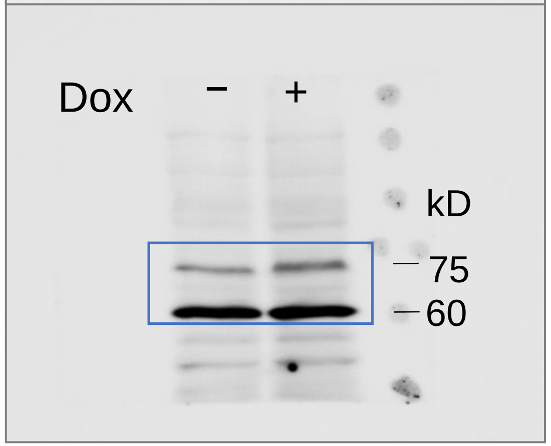

Fig 5C: pp70S6K 14d

Fig 5D

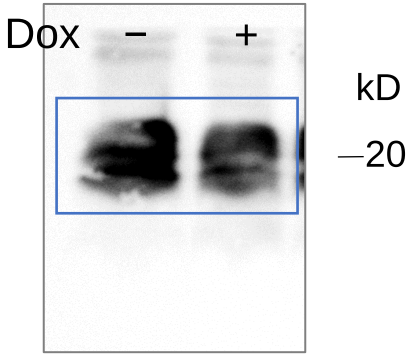

Fig 5D: p4EBP1 2d

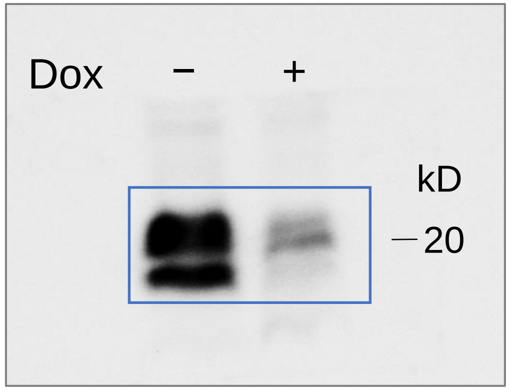

Fig 5D: p4EBP1 7d

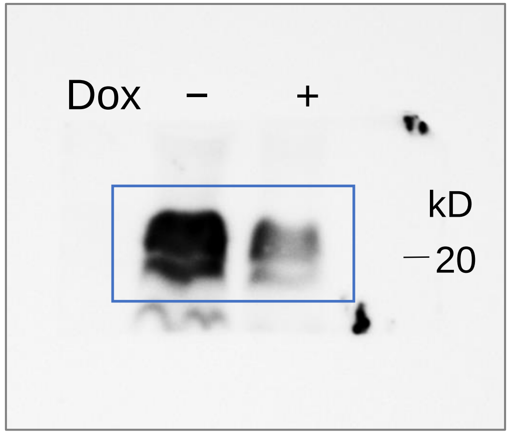

Fig 5D: p4EBP1 14d

S3 Fig

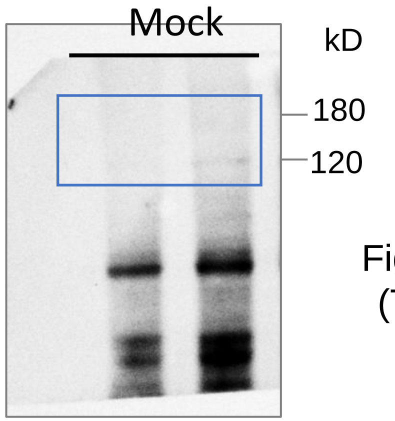

Fig S2B: COL17
(Tumor lysate)

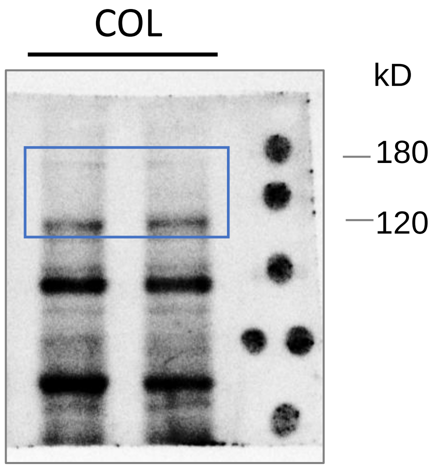

Fig S2B: COL17
(Tumor lysate)

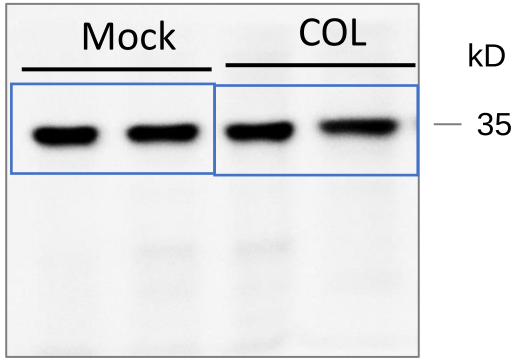

Fig S2B: β-actin
